# Multi-scale analysis of schizophrenia risk genes, brain structure, and clinical symptoms reveals integrative clues for subtyping schizophrenia patients

**DOI:** 10.1093/jmcb/mjy071

**Published:** 2018-12-03

**Authors:** Liang Ma, Edmund T Rolls, Xiuqin Liu, Yuting Liu, Zeyu Jiao, Yue Wang, Weikang Gong, Zhiming Ma, Fuzhou Gong, Lin Wan

**Affiliations:** 1 CAS Key Laboratory of Genomic and Precision Medicine, Beijing Institute of Genomics, Chinese Academy of Sciences, Beijing, China; 2 National Center of Mathematics and Interdisciplinary Sciences, Academy of Mathematics and Systems Science, Chinese Academy of Sciences, Beijing, China; 3 Department of Computer Science, University of Warwick, Coventry, UK; 4 Oxford Centre for Computational Neuroscience, Oxford, UK; 5 School of Mathematics and Physics, University of Science and Technology Beijing, Beijing, China; 6 School of Science, Beijing Jiaotong University, Beijing, China; 7 Centre for Computational Systems Biology, School of Mathematical Sciences, Fudan University, Shanghai, China; 8 CAS-MPG Partner Institute for Computational Biology, Shanghai Institutes for Biological Sciences, Chinese Academy of Sciences, Shanghai, China; 9 University of Chinese Academy of Sciences, Beijing, China

**Keywords:** Schizophrenia, PANSS, multi-scale analysis, hot cluster, grey matter volume, pathway

## Abstract

Analysis linking directly genomics, neuroimaging phenotypes and clinical measurements is crucial for understanding psychiatric disorders, but remains rare. Here, we describe a multi-scale analysis using genome-wide SNPs, gene expression, grey matter volume (GMV), and the positive and negative syndrome scale scores (PANSS) to explore the etiology of schizophrenia. With 72 drug-naive schizophrenic first episode patients (FEPs) and 73 matched heathy controls, we identified 108 genes, from schizophrenia risk genes, that correlated significantly with GMV, which are highly co-expressed in the brain during development. Among these 108 candidates, 19 distinct genes were found associated with 16 brain regions referred to as hot clusters (HCs), primarily in the frontal cortex, sensory-motor regions and temporal and parietal regions. The patients were subtyped into three groups with distinguishable PANSS scores by the GMV of the identified HCs. Furthermore, we found that HCs with common GMV among patient groups are related to genes that mostly mapped to pathways relevant to neural signaling, which are associated with the risk for schizophrenia. Our results provide an integrated view of how genetic variants may affect brain structures that lead to distinct disease phenotypes. The method of multi-scale analysis that was described in this research, may help to advance the understanding of the etiology of schizophrenia.

## Introduction

Schizophrenia is one of the most common psychotic disorders worldwide, with a lifetime prevalence of 0.3%–0.7% (van Os and Kapur, 2009). The age of onset of Schizophrenia is typically in late adolescence or early adulthood, with males more often affected than females (Gejman et al., 2010). The heritability of schizophrenia has been reported to range from 44% to 87% with a mean of 81% (Sullivan et al., 2003). Schizophrenia is increasingly recognized as a collection of syndromes as opposed to a single disease entity, and involves a number of cognitive and emotional impairments (Jablensky, 2006, 2010).

The advances in new generation sequencing approaches, neuroimaging technologies and a variety of clinical questionnaires have provided an unprecedented opportunity to understand psychiatric disorders like schizophrenia. For example, genome-wide association studies (GWAS) provided unique ways to explore the potential genetic causes of schizophrenia. Thousands of loci on the genome have been identified to be associated with schizophrenia (Allen et al., 2008), e.g. *ANK3*, *ZNF804A*, *CACNA1C*, *NRG1*, *TCF4*, and MHC region, etc (O’Donovan et al., 2008; Ripke et al., 2013; Schwab and Wildenauer, 2013). Schizophrenia is believed to be a polygenetic disease, and Gilman et al. identified gene networks from different types of schizophrenia-associated genetic variations (Gilman et al., 2012). Schizophrenia-associated genes harboring *de novo* mutations showed significant spatio-temporal co-expression patterns when mapped onto transcriptome profiles of normal human brain tissues (Gulsuner et al., 2013).

Neuroimaging studies based on magnetic resonance imaging (MRI) methodology have led to a better understanding of the neuroanatomical basis of schizophrenia. Structural brain alterations, in particular grey matter density or grey matter volume (GMV) in the whole brain or regions such as the hippocampus, parahippocampal gyrus, amygdala, thalamus, insular cortex, anterior cingulate, left middle frontal gyrus, and postcentral gyrus have been reported to be reduced in schizophrenic patients relative to healthy controls (Honea et al., 2005; Steen et al., 2006; Glahn et al., 2008; Gupta et al., 2015; van Erp et al., 2015). Twin, family or sibling-based studies have suggested that brain morphology characterized by neuroimaging quantitative measures is highly heritable (Sullivan et al., 2003; Thompson et al., 2010; Turner et al., 2012). The development of imaging genetics methods has leveraged the understanding of the possible genetic bases of brain structural differences and made it possible to map the genetic effects onto brain regions (Ge et al., 2013).

The symptoms of schizophrenia are heterogeneous and are mainly categorized into two major types, the positive symptoms and the negative symptoms. The positive symptoms, which represented as an excess or distortion of normal functions such as hallucinations and delusions. The negative symptoms which refer to the loss or diminution of normal functions, for example, absence in grooming, language or communication. The psychotic severity is often assessed by different rating scales, such as the positive and negative syndrome scale (PANSS), brief psychiatric rating scale (BPRS), scale for the assessment of positive symptoms (SAPS), or scale for the assessment of negative symptoms (SANS) (Overall and Gorham, 1962; Kay et al., 1987). Whitford et al. (2005) suggested that the symptom severity is associated with brain structural abnormality in schizophrenia. Nesvåg et al. (2009) reported that the positive and negative symptoms in patients with schizophrenia can be related to variations in cortical and grey matter volumes.

Despite the large number of investigations in genetics, structural and functional brain changes, and behavioral abnormalities of schizophrenia, there are currently few studies that identify simultaneously links through genetics, neuroimaging and behavior, where much correlations exhibit among these aspects. Existing approaches usually focus on one scale or across two scales, which lead to incomplete understanding of disease etiology. Therefore, a framework that harnesses multi-scale data, from genetics through endophenotypes, e.g. brain structure, to phenotypes such as symptom severity is crucial for understanding schizophrenia.

In this study, we describe a multi-scale analysis that utilizing data from genomics, transcriptomics, neuroimaging, and clinical measurements to explore the etiology of schizophrenia. We first established a link between genes and brain structural (grey matter) changes by association study using schizophrenia risk genes reported in the SZGene database (Allen et al., 2008) and structural MRI imaging of the brain. Genes that showed significant association with GMV were selected as candidates and further validated by analyzing their spatio-temporal co-expression patterns in normal human brain during development. The collective brain regions which associated with candidate genes were referred to as hot clusters (HCs). The mean GMV of the identified HCs was used to subtype patients, and the patients of different subtypes were found to have distinguishable PANSS scores. By this multi-scale analysis, we linked genetically associated brain structural alterations to the symptoms of schizophrenic patients. We hypothesize that the identified genes cause structural brain changes, which further lead to distinct behavioral expressions of schizophrenic patients. The integrated results across multi-scale data (i.e. genetic, neuroimaging, behavioral) are expected to provide a more complete understanding of the genetic and biological mechanisms underlying schizophrenia, and to shed light into how genetic variation and brain structural alterations are related to different symptom scores in patients with schizophrenia.

## Results

### Pre-processing of brain MRI data and selection of schizophrenia risk genes

A total of 145 Han Chinese subjects took part in this study, which were recruited from the Mental Health Center of West China Hospital, Sichuan University in China. These subjects including 72 drug-naive schizophrenic first episode patients (FEPs) and 73 heathy controls who were all matched with age, gender, and years of education (see Supplementary Table S1). The diagnosis of schizophrenia and duration of illness were determined by the consensus of the attending psychiatrist and a trained interviewer using the Structured Clinical Interview for DSM-IV (SCID-P), and the diagnoses of all patients were confirmed after at least one year of follow-up. Healthy controls were recruited from the local area through poster advertisement and screened using SCID-NP to confirm the lifetime absence of psychiatric and neurological illness. In addition, the control subjects were interviewed to ensure that there was no history of psychiatric illness in first-degree relatives. Brain structural MRI was obtained from each subject through identical scanner and imaging protocols. Genetic data for every participant were also collected. The study protocol was approved by the ethical committee of the West China Hospital of Sichuan University.

High-resolution T1-weighted images were acquired from samples on admission using a 3-Tesla MRI system (EXCITE, General Electric) and an eight-channel phase array head coil with a volumetric 3D Spoiled Gradient Recall (SPGR) sequence. The voxel-based morphometry (VBM) preprocessing of T1-weighted structural data was carried out using DARTEL Tools from the Statistical Parametric Mapping package (SPM8, http://www.fil.ion.ucl.ac.uk/spm) under MATLAB Release 2014a (The MathWorks, Natick, 2014). Each retained image had a total of 433584 voxels (with size: 1.5 mm^3^) for further statistical analysis. The automated anatomical labeling (AAL) (Tzourio-Mazoyer et al., 2002) atlas, which partitioned the brain into 90 regions of interest (ROIs; 45 in each hemisphere), was used to identify the brain regions (see Supplementary Text S1 for more details).

The Genetic data were extracted by performing the HumanOmniZhongHua-8 Bead Chip (Illumina). Participants with a low genotyping rate (<97%), markers with >5% missing data and markers with minor allele frequency (MAF) ≤0.05 (calculated based on both case and control samples) or markers that failed to pass the Hardy–Weinberg equilibrium tests (*P* ≤ 10^−6^) were excluded from further analysis.

The SZGene database identified 994 schizophrenia risk genes reported from various genome-wide association studies, including both protein-coding and non-coding genes. Only protein-coding genes reported by the SZGene database (http://www.szgene.org/) which also appeared in the expression data list of BrainSpan database (http://www.brainspan.org/) were considered in this study. After mapping all SZGene genes to the selected genes from the BrainSpan dataset (see below), and after quality control, 718 schizophrenia risk genes with 20982 SNPs were retained for further analysis. Note that the SNPs are mapped to the genes according to the annotation on Ensembl database. The SNPs within the range of a gene are annotated by the gene symbol. We did not distinguish between the exon or intron types of the SNPs in this study.

### Association between risk genes and grey matter variation

We assessed the associations of the 718 protein-coding genes that convey risk for schizophrenia with voxel-wise brain structural variation (e.g. GMV). Of all 718 genes, 108 genes remained significant and were selected as candidates, including 246 SNPs in association with 6103 voxels. The top 20 significant SNPs ranked by minimum *P*-values are given in Supplementary Table S3 and the mapped locations within the brain regions according to the top four significant SNPs are displayed in Supplementary Figure S1.

### The 108 candidate genes are highly co-expressed in a spatio- and temporal-specific manner

**Figure 1:**
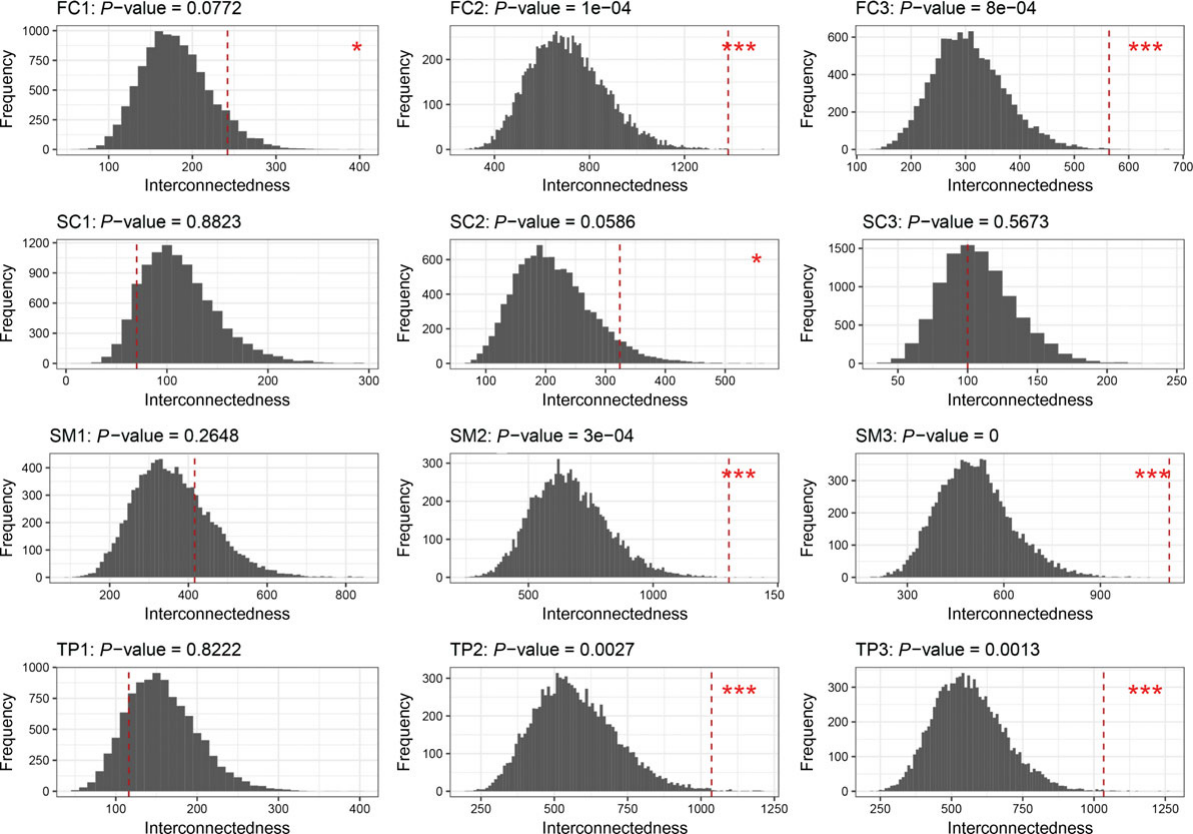
The *P*-values of the interconnectedness of the 108 candidate genes in the 12 spatial-temporal brain developmental gene networks. The subplots of each row correspond to four anatomical regions—frontal cortex (FC; first row), subcortical regions (SC; second row), sensory-motor regions (SM; third row); and temporal and parietal regions (TP; fourth rows). The subplots of each column showed the three brain developmental stages— fetal (period 1: 8–37 post-conception weeks; front column), early infancy to late childhood (period 2: 4 months to 8 years; middle column); and adolescence to adulthood (period 3: 13–40 years; last column). Each subplot presents the empirical distribution of interconnectedness of 108 genes which drawn randomly from 718 schizophrenia risk genes. The empirical distributions were generated by resampling 10000 times at each spatial and temporal period. The red vertical dashed line in each subplot shows the interconnectedness of the 108 candidate genes. The *P*-values of the interconnectedness of the candidate genes were calculated as the fraction of the interconnectedness of 10000 sets larger than the observed interconnectedness (corresponding to column “p-0.8w” of Supplementary Table S4). The spatio-temproal networks with 108 candidate genes presented significant at *P*-value < 0.01 were annotated with *** and at *P*-value < 0.1 were annotated with *.

We constructed 12 spatio-temporal gene co-expression networks from 718 risk genes accounting for four anatomic brain regions across three developmental stages (Supplementary Table S2). The empirical distributions of interconnectedness of 108 randomly sampled genes (out of the risk genes) in these 12 networks are shown in Figure 1. We found that the distribution of interconnectedness of the candidate genes changes with different developmental stages. During the early infancy to late childhood stage and the adolescence to adulthood stage, the candidate genes showed significantly stronger interconnectedness in networks corresponding to the frontal cortex (FC) region, the sensory-motor regions (SM), and the temporal and parietal regions (TP) of brain (*P*-value < 0.01). The interconnectedness of candidate genes was also stronger in the FC region during the fetal period and in the subcortical regions (SC) during the adolescence to adulthood stage (*P*-value < 0.1) (Figure 1 and Supplementary Table S4).

Stability tests showed that the significances of interconnectedness under different thresholds and weighting strategies were robust (Supplementary Table S4). The interconnectedness of candidate genes also showed no significant result in networks constructed from 718 genes by merging all spatial and temporal samples from BrainSpan (the last row of Supplementary Table S4 shows the results of merged BrainSpan samples). No significant results were obtained when candidate genes were tested using protein–protein interaction from GeneMANIA (Mostafavi et al., 2008) (*P*-value=0.318).

### Nineteen candidate genes are associated with 16 grey matter HCs in the brain

**Figure 2:**
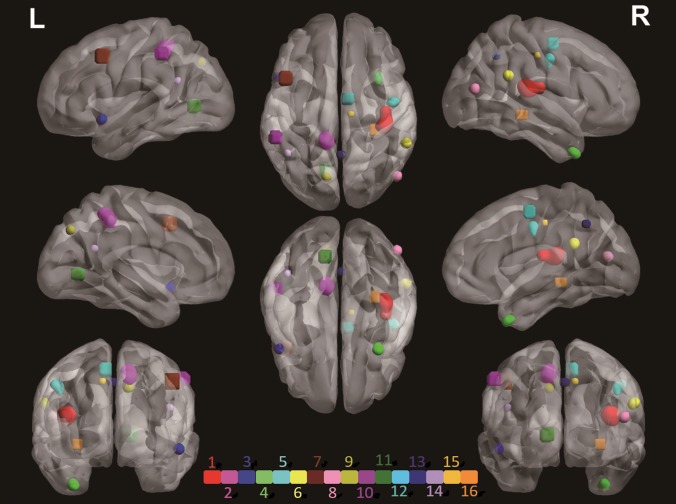
3D image of the 16 HCs identified. The HCs were denoted as “HC1”, “HC2”,…, “HC16” according to Table 1. Each HC is represented with a unique color. The coronal views of each cluster are shown in Supplementary Figure S2, with intensity representing the corrected weight of each voxel. The exact location and the related genes of each HC are provided in Table 1 and Supplementary Table S5.

Connected brain regions that were collectively associated with candidate genes were identified as HCs (for details of these genes and HCs, see Table 1 and Supplementary Table S5). We observed that among the 108 candidate genes there are 19 different genes, which we refer as to HC genes, associated with 16 HCs, where the HC1–HC16 were named according to the decreased rank of the maximum voxel weight of each HC. Figure 2 shows the locations of the HCs in 3D view and the coronal view of each HC is shown in Supplementary Figure S2 with intensity representing the corrected weight of each voxel. The cluster with the maximum single voxel weight is HC1, which also formed the largest cluster with a total of 945 voxels. It is associated with genes *CACNA1A* or *ERBB4* and located in the right insula (INS.R) region. HC2 is in association with *PPP1R1B*. It incorporates 438 voxels that lie in the left precuneus (PCUN.L). HC3 lies in the left middle temporal pole (TPOmid.L) with 118 voxels and is related to *ATP2B2*. HC4 is in association with genes *NRG3* and *PPP3C*. It located in the right middle temporal pole region (TPOmid.R). HC5, which is associated with *PPP3CA* and *RELN*, locates across the right precentral region (PreCG.R). Table 1 and Supplementary Table S5 show more information on the identified HCs.

**Table 1:** HCs and associated genes.

	Associated genes	AAL region	Brain region*	Cluster size
**HC1**	*CACNA1A*	Insula_R	SM	945
	*ERBB4*			
**HC2**	*PPP1R1B*	Precuneus_L	TP	438
**HC3**	*ATP2B2*	Temporal_Pole_Sup_L	TP	118
**HC4**	*PPP3CA*	Temporal_Pole_Mid_R	TP	134
	*NRG3*			
**HC5**	*ZBTB20*	Precentral_R	SM	157
	*RELN*			
**HC6**	*OPCML*	SupraMarginal_R	TP	74
**HC7**	*INPP4B*	Frontal_Mid_L	FC	63
**HC8**	*ZNF365*	Temporal_Mid_R	TP	44
**HC9**	*ANK3*	Precuneus_L	TP	47
**HC10**	*ZBTB20*	Parietal_Inf_L	TP	31
**HC11**	*PSAP*	Lingual_L	OC	33
**HC12**	*BMP6*	Supp_Motor_Area_R	SM	20
	*EIF2B5*			
**HC13**	*NRG3*	Precuneus_R	TP	16
	*PRKCA*			
**HC14**	*FGF1*	Angular_L	TP	11
**HC15**	*SLIT3*	Cingulate_Mid_R	FC	8
**HC16**	*GFRA2*	Hippocampus_R	SC	11

The HCs and HC genes. The number of voxels contained in the HCs is listed in the last column. More details can be found in Supplementary Table S5.

*FC, frontal cortex; TP, temporal and parietal regions; SM, sensory-motor regions; SC, subcortical regions; OC, occipital regions.

The HC genes are found to be mapped to neuro signaling and related pathways (Supplementary Figure S3). Genes *CACNA1A*, *ERBB4*, *ATP2B2*, *PPP3CA*, and *PRKCA* that affect HC1, HC3, H4, and H13 can be related to the calcium signaling pathway (Supplementary Figure S3A). *CACNA1A* encodes P/Q-type calcium channels, which are the targets of Dopamine D2 receptors for antipsychotic drugs (Galizzi et al., 1986; Nimmrich and Gross, 2012). The cAMP pathway, where the *PPP1R1B* gene of HC2 is located, is the interconnecting node for the Dopamine D2 receptor cascade of the P/Q-type calcium channels (Greengard et al., 1999). Gene *INPP4B* (HC7) was found mapped to the phosphatidylinositol signaling pathway, which locates downstream to the calcium signaling pathway (Supplementary Figure S3A). Genes *ERBB4*, *NRG3*, and *PRKCA* are mapped to the ERBB signaling pathway which is located upstream of the calcium signaling pathway (Supplementary Figure S3B). Recent studies provided strong evidence that the calcium and ERBB signaling pathways are associated with schizophrenia (Cross-Disorder Group of the Psychiatric Genomics, 2013; Harrison, 2015). Mutations of the *ERBB4* gene differentially affected the treatment response to paliperidone in individuals with schizophrenia, implicating the neuregulin 1 (NRG1)–ErbB4 pathway for modulating the antipsychotic response (Wang et al., 2015). *ERBB4* is activated directly by gene *NRG3* (related to HC4 and HC13). The *ERBB4* gene activates the downstream PI3K–Akt signaling pathway which gene *RELN* (HC5) and *FGF1* (HC14) mapped to (Supplementary Figure S3B) (Law et al., 2012). *RELN* is a key gene for neuronal migration during brain development. Mutation of the *RELN* gene in the mouse results in motor problems (Sanes et al., 2012). A lack of *RELN* causes a form of lissencephaly (Hong et al., 2000). Further, the Neuregulin1–ErbB4–PI3K signaling has been shown to be a schizophrenia risk pathway, with potential therapeutic relevance (Law et al., 2012). Genes *CACNA1A*, *PPP1R1B*, *PPP3CA*, and *PRKCA* are related to the Dopaminergic Synapse pathway (Supplementary Figure S3C). Genes *CACNA1A*, *PPP3CA*, and *PRKCA* also function in the Glutamatergic Synapse pathway (Supplementary Figure S3D). Dopaminergic and glutamatergic systems are closely related in the brain: dopaminergic activity can inhibit glutamatergic function and can result in a hypoglutamatergic state in schizophrenia (Kötter, 1994; Bradford, 2009).

### Patients subtyped according to GMV of HCs show differences in positive/negative symptom scale

The symptoms of schizophrenia patients can be heterogeneous with two main types, namely, the positive symptoms and the negative symptoms. These symptoms are related to the variation in cortical and grey matter volumes of schizophrenia patients (Nesvåg et al., 2009). We have 67 schizophrenia patients with PANSS scores. We thus hope to subtype the patients according to their grey matter variations of HCs and further characterize their symptoms.

**Figure 3:**
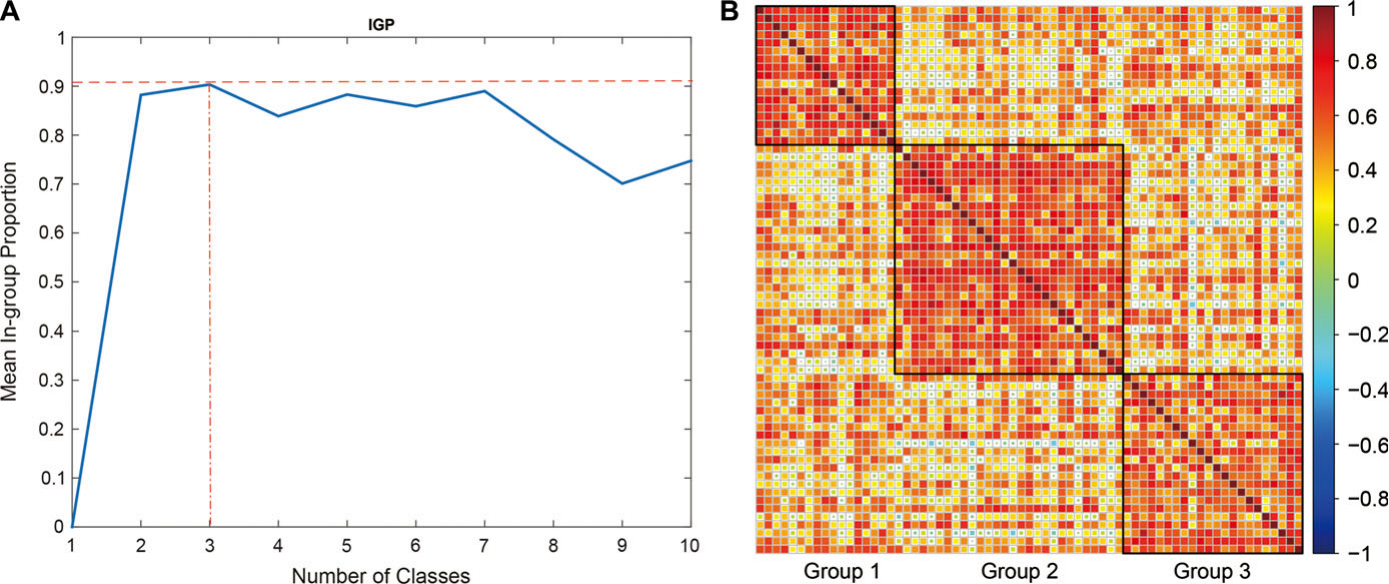
Identification of subtype patients by GMV of HCs. (**A**) The number of classes against mean in-group proportion (IGP). The optimum number of classes (groups) is identified by maximizing IGP. (**B**) Heat map of the three groups identified according to grey matter densities (GMVs) of the 16 HCs. The patients were sorted by groups. The colors represent the correlation coefficient (as displayed in the color bar) of GMVs over 16 HCs of two corresponding patients.

We have 67 schizophrenia patients with PANSS scores and they were subtyped by *K*-means method according to the GMV of HCs. The optimum number of groups was set to 3 according to the index of in-groups proportion (Kapp and Tibshirani, 2007) (IGP) (Figure 3A). Figure 3B shows the GMV correlation map of the three groups of clustered patients. The sizes of the groups are 17, 28, and 22, respectively, with no significant difference in gender and age composition (data not show). As shown in Supplementary Figure S4A, patients in Group 1 have higher mean scores on 5 of 7 items in the positive PANSS symptoms, while Group 2 patients showed more items with higher mean negative PANSS symptom scores (5/7). All three group patients had their mean scores intermixed with each other on items in the general PANSS psychopathology scale (Supplementary Figure S4A).

**Figure 4:**
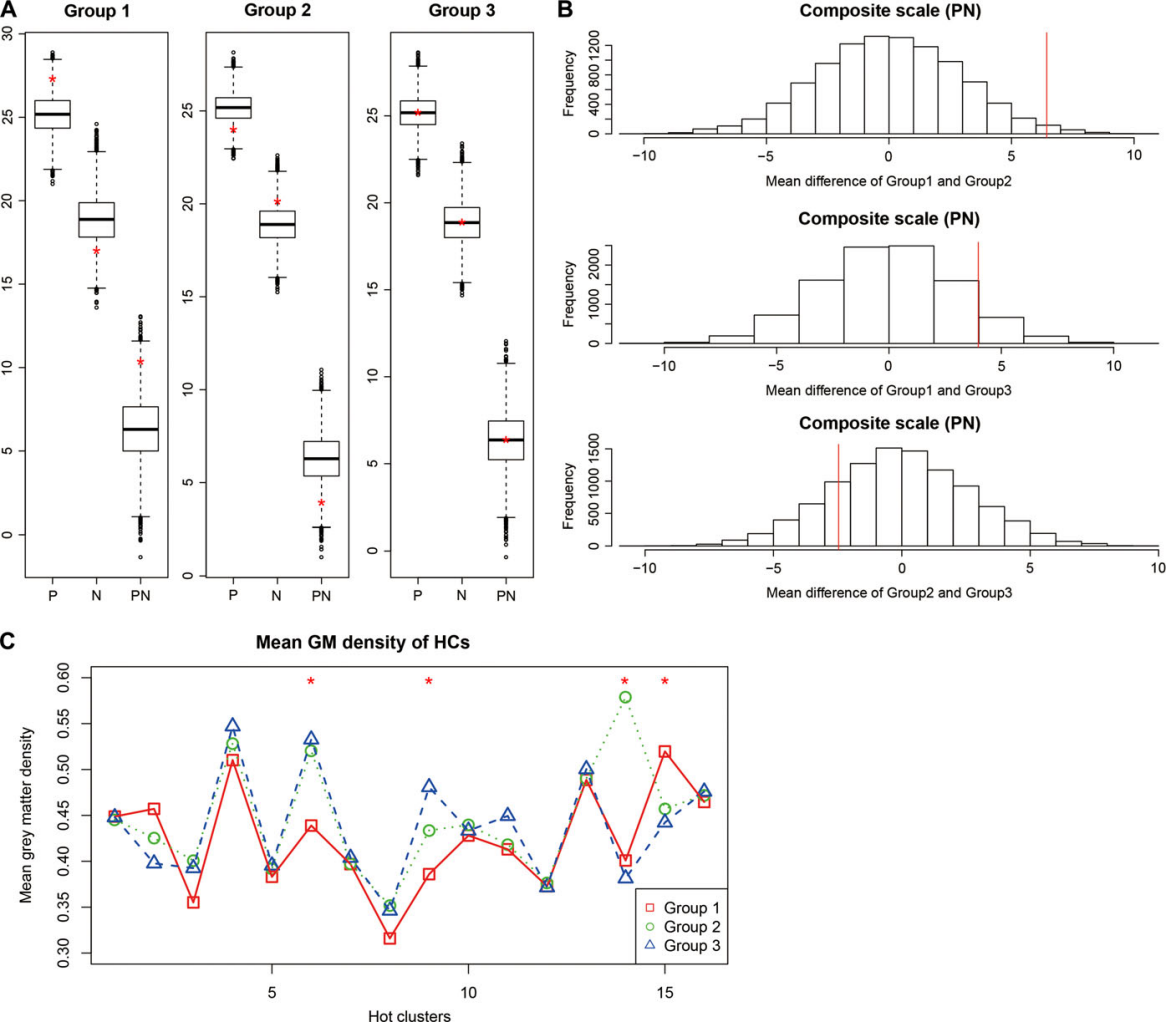
Positive and negative scores in different subtyped patients. (**A**) Boxplots of mean positive (P), negative (N), and composite (PN) scale scores over 10000 permutations. The red ‘*’ represents the mean score of the original un-permuted groups. (**B**) The empirical distribution of the Group-wise difference over 10000 permutations. The mean difference of the composite scale (PN) scores between Groups 1 and 2 (MeanPN12), Groups 1 and 3 (MeanPN13), and Groups 2 and 3 (MeanPN23) over 10000 permutations are shown in histograms. The x-axis represents the difference of group mean scores of the pair of permutated groups. The quantile of the *P*-value is represented by red lines. The x-axis represents the difference of the mean composite scale scores of a pair of permuted groups. The quantile of *P*-value is represented by red lines. (**C**) The mean group GMV of each HCs, with Group 1 shown in red, Group 2 in green and Group 3 in blue. The red ‘*’ indicate significant differences in GMV of the corresponding HC among groups (Supplementary Table S6).

We tested the differences in the subscales of PANSS between groups by random permutation of patients’ group labels. The three groups showed similar means in the total score (TT) and general psychopathology score (G) (Supplementary Figure S4B and C). Group 1 had a relatively higher positive score (P) and lower negative score (N), and thus, a significantly larger composite score, which defined by subtracting the negative from the positive score and we designated as PN for short (mean PN = 10.3, *P* < 0.05; PN are 3.9 and 6.3 in Groups 2 and 3, Figure 4A). In contrast, an opposite trend was observed for Group 2 (Figure 4A). When compared pairwisely, the PN differences between Groups 1 and 2 and between Groups 1 and 3 were also significant (*P*-values < 0.05 and < 0.1, respectively, Figure 4B).

Figure 4C shows the mean GMVs for each HC within patient groups. We conducted Wilcoxon rank sum tests to evaluate the mean GMV difference of HC between patients in one group versus the remaining. For each group, the *P*-values were corrected by Bonferroni method over the 16 HCs (*P*-values < 0.05/16). The results show that on HC6, HC9, and HC15, patients of Group 1 had significantly different GMVs compared to those of the remaining patients (Supplementary Table S6). Group 2 patients had mean GMV varied from the others on HC14, and Group 3 patients had significantly different GMVs on HC9 and HC14.

## Discussion

In this investigation, we describe a multi-scale analysis across data of multiple scales to identify clusters of attributes related to schizophrenia. We identified 108 candidate genes from 718 schizophrenia risk genes that exhibit association with GMV of the brain. These candidate genes are highly co-expressed in FC and TP during the early infancy to childhood period, and in SM through the early infancy to adulthood period. Among these 108 candidate genes, 19 distinct genes were further identified to be associated with the GMV of 16 HCs in the brain which were also mainly located in FC, TP, and SM regions. Recent studies have suggested that subtypes of schizophrenic symptoms may be linked to specific patterns of structural brain alterations (Koutsouleris et al., 2008; Nenadic et al., 2010; Zhang et al., 2015). Our results show that the identified HCs may serve as multidimensional traits which are able to characterize important features of the structural variations of schizophrenia patients that manifest in distinguishable symptoms. The patients are subtyped into three groups by using GMV of 16 HCs, with patients in Group 1 showing more severe positive symptoms and patients of Group 2 showing higher negative symptoms scores.

In this study, the HCs were identified by genes with variations highly associated to the grey matter variation of these regions. It does not mean that these genes are restricted to function in the HCs. Since schizophrenia is a developmental disorder, the HCs can be regarded as the initial points of schizophrenia that have grey matter abnormality. With the development of schizophrenia, these initial points will spread out (like a diffusion processes). All of these do not conflict with the view that gene expression in the brain is widespread. In fact, as the results in our spatio-temporal expression analysis, the candidate genes are highly co-expressed in a core region of human (FC) at first, and spread to co-express in most brain regions as developmental progresses. Here we found that schizophrenia has multiple initial points (HCs), and different HCs are shown to be associated with different subtypes of schizophrenia.

More interestingly, the genes related to HCs that show little mean GMV difference between the three patient groups, such as HC1, HC5, HC7, HC10, HC12, HC13, and HC16, are mainly mapped to KEGG pathways relevant to neural signaling (Supplementary Figure S3). These pathways are known to be most basic in neurodevelopment (Karam et al., 2010; Berwick and Harvey, 2014; Wu, 2017). We hypothesize that these HCs carry common GMV variations that contribute to all schizophrenic patients.

The differed symptoms, on the other hand, might be contributed by HC6, HC9, HC14, and HC15, which show much variance among patient groups. In detail, Group 1 patients had significantly reduced mean GMV as compared to patients of other groups in HC6. The HC6 located in the left supramarginal region, which is related to language perception and processing. In HC15, which located in the right median cingulate and paracingulate gyri, patients of Group 1 had a larger mean GMV. This region is implicated in error detection, attention or motivation-related functions and is reported to be associated with schizophrenia (Posner and DiGirolamo, 1998). Group 2 patients had a greater mean GMV on HC14, which located in the left angular gyrus and associated with language. The three groups have quite distinct mean GMVs on HC9, where Group 1 patients have the lowest mean GMV, and Group 3 patients have a significantly larger mean, with the mean GMV of patients of Group 2 in between. HC9 located across the left cuneus, superior occipital gyrus, superior parietal gyrus, and precuneus regions. These regions are involved in visual processing, spatial orientation, episodic memory, visuospatial processing, reflections upon the self, and aspects of consciousness. In particular, the precuneus, where HC2 and HC13 were also located, along with adjacent areas within the posteromedial parietal cortex, has attracted increasing attention, as it is among the most active cortical regions according to the ‘default mode’ of brain function during the conscious resting state, whereas it selectively deactivates in a number of pathophysiological conditions and neuropsychiatric disorders, including schizophrenia, characterized by impaired information processing (Cavanna, 2007).

Finally, there are several remarks we would like to address. First, our goal was to search for potential collective effects of multiple genes rather than looking for new genetic locations correlated with brain structural variation. The SZGene database which we used to extract schizophrenia risk genes, although stopped updating, includes the largest collection of schizophrenia-related genes to our knowledge. However, our method can be easily applied to an extended list of genes. Second, the transcriptional profiles obtained from BrainSpan are non-psychiatric postmortem donors. This study is therefore limited to examining the co-expression pattern of candidate genes within the group of putatively normal persons. We believe that, with the accumulation of transcriptome samples on psychiatric patients in the future, examining the possible cis- and trans- effects on variants, and in turn the gene expression characteristics in specific HCs will strengthen the understanding of links between the genetics and the symptoms of schizophrenia (Bray et al., 2008; Jiang, 2015; Wu et al., 2017). Third, studies of schizophrenia are more increasingly interested in using functional MRI data (Gong and Lin, 2018). Our findings revealed that the collective effects of candidate genes on HCs of SCZ patients may result in different symptoms as characterized by the PANSS score. However, how these HCs are functionally connected or how they are related to other regions calls for further investigation. Last, the symptoms of patients, which subtyped into three groups according to the HCs, are distinguishable by the scores on the positive and negative items of PANSS in this study. It is expected that with a larger size of samples together with multi-scale data, a more refined classification of patients may be possible.

## Materials and methods

### Brain developmental gene expression data

The BrainSpan database contains the largest transcriptome profiles of human brain development with up to 16 cortical and subcortical structures across the full course from post-conception weeks (PCWs) to adulthood. We used this data to evaluate the spatial-temporal co-expression of selected candidate genes. The gene expression data of normal human brain tissues were from BrainSpan database. After filtering out non-coding genes, and genes with low expression level, a total of 15272 genes were retained as background for further co-expression analysis (see Supplementary Text S2).

### Identification of candidate genes using the linear regression model

A linear regression model (Gong et al., 2014) was used to detect the association between single gene/SNP and the GMV of voxels in the brain, with age, gender and education as covariates (Supplementary Text S3). A risk gene was selected as a candidate if at least one SNP of the gene had a minimum *P*-value < 1 × 10^−6^ (see Supplementary Text S4 on the discussion of false discovery rate controls). The analysis was performed using MATLAB Release 2014a (The MathWorks, Natick, 2014).

### Construction of spatio-temporal co-expression gene networks

The BrainSpan dataset was divided into 12 sub-sets according to 4 anatomical regions of the brain and 3 predefined developmental stages (Supplementary Table S2). In particular, the brain was partitioned into non-overlapping regions: FC, TP, SM, and SC; and samples were categorized into developmental stages according to their ages: fetal (8–37 post-conception weeks), early infancy to late childhood (4 months to 8 years), and adolescence to adulthood (13–40 years) (Gulsuner et al., 2013) (see Supplementary Table S2 for more details). For each sub-set, the absolute Pearson correlation coefficients (denoted as PCC) of expression of each pair of the schizophrenia risk genes were calculated. As strong correlation of expression between a pair of genes may potentially indicate high relevance to function, we applied a common threshold of PCC > 0.8, and set values below the threshold to 0. Twelve spatio-temporal specific weighted gene co-expression networks, each with risk genes as nodes and edges assigned with threshold PCCs, were thus constructed. The co-expression strength of a set of genes was characterized by interconnectedness, which was defined as the sum of the edges’ weights among the set of genes.

We applied a resampling procedure to test if the candidate genes are highly interconnected. The stabilities of the results were also evaluated by comparing different PCC thresholds (PCC=0.8, unweighted networks and merged networks (Supplementary Text S5). The analysis was performed using R environment (https://www.R-project.org/).

### Construction of grey matter HCs based on collective genetic effects

Since many common genetic variants of small effect may contribute to the variation of GMV, we clustered the voxels with GMV closely associated with a number of candidate gene/SNPs. All voxels were first weighted by the number of SNPs which were significantly associated with their GMV. The weights of the voxels were further corrected taking into consideration the linkage disequilibrium (LD) among SNPs that are physically located on the same chromosome (Supplementary Text S6) and spatially smoothed by an isotropic Gaussian kernel (full-width at half-maximum=8 mm). Voxels with weight exceeding a pre-defined threshold of 0.2304 (Supplementary Text S7) were merged to form clusters based on a connectivity rule, that is, voxels sharing at least one edge or side. We named these identified clusters as HCs.

### Subtyping schizophrenic patients by HCs

We applied the *K*-means clustering approach using GMV of the HCs to divide the 67 patients that PANSS scores (Kay et al., 1987) into different groups. For each patient, the GMV of HCs was extracted and used as multidimensional features. The metric used for clustering was unity minus the correlation of the corresponding multidimensional features. In-group proportion (IGP; Kapp and Tibshirani, 2007), which quantifies the proportion of samples in a group whose nearest neighbors are also in the same group, was adopted to measure the quality of the clustering results.

The optimum number of groups (clusters) was determined according to the average IGP of all groups (the numbers of groups tested were from 2 to 10). For each number of groups chosen, 1000 replicate runs were conducted with locations of the centroid for each group initiated randomly. The sum of the pointwise distances to their centroid within groups was calculated for each replicate, and the minimum sum was chosen as the final outcome.

The total PANSS score (TT), and four subscales, namely, the positive scale score (P), the negative scale score (N), the general psychopathology scale score (G), and the composite scale score (defined by subtracting the negative from the positive score, i.e. PN), were calculated for each patient. The mean scores were compared across groups for each subscale by random permutation of the group label of patients for 10000 times. The *P*-values were obtained as the percentile where the true value lies in the empirical distribution from 10000 permutations. All analyses were performed using R environment (https://www.R-project.org/).

## Supplementary Material

mjy071_Supporting_Information_final_mjy071

mjy071_Supp

## Notes

**Edited by Luonan Chen**
