# Supplementary material for: Multi-scale analysis of schizophrenia risk genes, brain structure, and clinical symptoms reveals integrative clues for subtyping schizophrenia patients
Source: J Mol Cell Biol. 2018 Dec 3;11(8):678–87. doi: 10.1093/jmcb/mjy071 (PMC6788727; doi:10.1093/jmcb/mjy071)
Supplement: mjy071_Supporting_Information_final_mjy071 [file mjy071_supporting_information_final_mjy071.docx]

**Supporting Information**

**Text S1** Imaging Data Acquisition and Preprocessing.

**Text S2** Brain developmental gene expression data processing.

**Text S3** Identification of candidate genes/SNPs using the linear regression model.

**Text S4** Analysis of FDR of the candidate SNPs.

**Text S5** Permutation test for interconnectedness and Stability tests.

**Text S6** Adjusting the linkage disequilibrium (LD) effects of SNPs for voxel weights.

**Text S7** Defining the threshold of the weights of the voxels that form a hot cluster.

**Table S1** Summary of the demographic characteristics of the participants.

**Table S2** Basic information on the BrainSpan Dataset.

**Table S3** Top 20 significant SNPs from the voxel-wise association study.

**Table S4** The *p*-values of the interconnectedness of the 108 candidate genes in the spatial-temporal brain developmental gene networks under different construction strategies.

**Table S5** The 16 hot-clusters identified (ranked according to the “Maximum Peak” in the last column).

**Table S6** Grey matter volume difference of hot clusters.

**Table S7** All Candidate SNPs.

**Figure S1** Slice views of the top 4 significant SNPs and genes with their associated location in brain.

**Figure S2** Coronal views of the 16 hot clusters (HCs).

**Figure S3** The hot cluster genes within pathways.

**Figure S4** PANSS of different subtyped patient groups.

**Figure S5** FDR distribution of candidate SNPs.

**Figure S6** Thresholding weights of voxels.

**Supplementary Information**

**Text S1** Imaging Data Acquisition and Preprocessing

High-resolution T1-weighted images were acquired from samples on admission using a 3-Tesla MRI system (EXCITE, General Electric, WI, USA) and an eight-channel phase array head coil with a volumetric 3D Spoiled Gradient Recall (SPGR) sequence. The parameters for this imaging were as follows: repeat time (TR), 8.5 mm; echo time (TE), 3.4 mm; flip angle (FA), 12°; slice thickness (ST), 1 mm; NEX value, 1; field of view (FOV), 240×240 mm^2^; matrix size (MS), 256×256; generating 156 contiguous axial slices with the in-plane resolution of 0.47 mm × 0.47 mm^2^[25, 26].

The voxel-based morphometry (VBM) preprocessing of T1-weighted structural data was carried out using DARTEL Tools from the Statistical Parametric Mapping package (SPM8, http://www.fil.ion.ucl.ac.uk/spm). A quality control step was carried out first by visually checking and manually adjusting all T1-weighted structural data to ensure they were in a suitable format for further processing. The “new segment option” was then selected for the segmentation of the images and identification of the grey and white matter. Next, the grey matter images were aligned to a nonlinear deformation field and normalized to MNI space. Finally the normalized images were all smoothed according to an isotropic Gaussian kernel (full-width at half-maximum = 8 mm). After the above procedure, the spatially normalized, smoothed and Jacobian scaled grey matter images (voxel size: 1.5*1.5*1.5 mm, each image had 2,122,945 voxels) were obtained for each subject. Since voxels with low amount of grey matter have variances close to zero and do not apply to the Gaussian error distributions, we masked those low valued voxels with the SPM Masking Toolbox (<http://www0.cs.ucl.ac.uk/staff/g.ridgway/masking/>) with a software suggested threshold of 0.221761 calculated by SPM. As a result, each retained image had a total of 433,584 voxels for further statistical analysis. The automated anatomical labeling (AAL)[27] atlas, which partitioned the brain into 90 regions of interest (ROIs; 45 in each hemisphere), was used to identify the brain regions.

**Text S2** Brain developmental gene expression data processing

We downloaded the brain developmental gene expression data from *http://www.brainspan.org/api/v2/well_known_file_download/267666525* (“RNA-Seq Gencode v10 summarized to genes”). This dataset contains 52,376 transcripts (52,376 unique Ensembl IDs corresponding to 47,808 unique official gene symbols) across 524 tissue samples (42 normal subjects; 26 brain tissues; 31 developmental periods ranging from 8 PCWs to 40 years).

The transcripts were filtered by the following two procedures. First, we filtered out non-coding genes, with 19,672 protein coding genes retained based on GRCh37 annotation (Ensembl release 75 from BioMart, with 22,835 protein coding genes included). We then filtered out protein coding genes with low expression level in the BrainSpan dataset, with 15,272 genes remaining which have 80% of their reads per kilo million bases (RPKMs) greater than 0 and at least one RPKM greater than 1 across all samples in the BrainSpan dataset.

**Text S3** Identification of candidate genes/SNPs using the linear regression model

A linear regression model was used to detect the association between a single gene/SNP and the grey matter volume (GMV) of a voxel,

$$\text{Y}(\nu)=\text{X}\beta(\nu)+\varepsilon(\nu),$$

where$\nu$indicates the index of a voxel and $\text{Y}(\nu)=[Y_{1}(\nu),Y_{2}(\nu),...,Y_{N}(\nu)]'$ is the vector of GMV on voxel $\nu$ across *N* subjects; $\text{X}$is an N by 5 design matrix, where the first 4 columns include the intercept and 3 covariates: age, gender and education, respectively. The last column of $\text{X}$contains the number of minor alleles of the SNP under investigation; $\beta(\nu)$is a 5-dimension vector of unknown parameters, and $\varepsilon(\nu)$ denotes an *N*-dimension vector of the random errors that are assumed to be independently and identically normally distributed following$N(0,\sigma(\nu))$. The residuals and the estimates of their variance can thus be written as,

$$\begin{matrix} e(\nu)=\text{Y}(\nu)-\text{X}\hat{\beta}(\nu), \\ \hat{\sigma}(\nu)=\frac{1}{f}e'(\nu)e(\nu), \end{matrix}$$

where$\hat{\beta}(\nu)$is the unbiased estimate of$\beta(\nu)$and $f=N-5$is the degrees of freedom. The t-statistic corresponding to the genetic effect at voxel$\nu$can be defined as,

$$T(\nu)=\frac{c'\hat{\beta}(\nu)}{\hat{\sigma}(\nu)\sqrt{c'(\text{X'X)}^{-1}c}},$$

where$c=[0,0,0,0,1]'$ extracts the column of SNP information.

**Text S4** Analysis of FDR of the candidate SNPs

The threshold was set to an uncorrected *p*-value of < 1× 10^-6^ in our single SNP and single voxel linear regression model. To give a general view of the false discovery rate (FDR) the candidate SNPs may have, we approximated their FDRs using the Benjamini-Hochberg algorithm for each candidate with 433,584 tests on the voxels separately (Supplementary Figure S5). We found that 98.09% of our significant tests (*p*-value<10^-6^) have FDR<0.05. There were some outliers, and the largest FDR may reach a value of 0.424. Nevertheless, we rounded our threshold and kept it to *p*-value<10^-6^.

**Text S5** Permutation test for interconnectedness and Stability tests

An empirical distribution, by resampling 10,000 times, of interconnectedness of candidate genes randomly drawn out of the 718 schizophrenia risk genes was calculated for each spatial and temporal network. The *p*-value of the interconnectedness of candidate genes was calculated as the fraction of the interconnectedness in 10,000 pseudo-samples larger than the observed interconnectedness of candidate genes. The stabilities of the results were evaluated by setting different thresholds (|PCC|=0.8, 0.7 and 0.6) and by comparing both unweighted (with 1 assigned to edges that exceed the thresholds), and weighted |PCC| networks. For comparison, we evaluated candidate genes over a network that merged samples from all spatial and temporal data (the merged network).

**Text S6** Adjusting the linkage disequilibrium (LD) effects of SNPs for voxel weights

Due to linkage disequilibrium (LD) of SNPs, weights (significant association with candidate genes/SNPs) of voxels may be over-calculated. Therefore we carried out a correcting strategy on the number of significant associations accounting for LD. We calculated the LD correlation coefficients R^2^ between SNPs on the same chromosome. The weight of a voxel calculated from each SNP was multiplied by 1/(n+1), where n is the number of other candidate SNPs on the same chromosome that have LD correlation coefficient R^2^ > 0.8 with respect to the SNP of interest. The final weight of each voxel was calculated as the weighted sum of all significant associations.

**Text S7** Defining the threshold of the weights of the voxels that form a hot cluster

The corrected weight of each voxel was compared with the weights of its neighbors (e.g. 26 neighbor voxels, e.g. the center of 3*3*3 voxels cubic), and voxels that showed a greater weight than all neighbors were selected. Voxels thus identified were labeled (local) peak voxels and ranked according to their corrected weights (i.e. number of associated SNPs). We define the adjacency differences as the differences of weights between peak voxels with adjacent rank. The adjacency differences were further ranked. Peaks voxels involved in the top 5% adjacency differences were selected, and the minimum weight of which is then set as the threshold (Supplementary Figure S6).

**Table S1** Summary of the demographic characteristics of the participants

|  | **Sample Size** | **Sex(M/F)** | **Age** | **Education** |
| --- | --- | --- | --- | --- |
| **Patient** | 72 | 37/35 | 24.2±7.5 | 12.3±3.5 |
| **Control** | 73 | 38/35 | 26.5±13.1 | 13.4±3.2 |
| ***p*-value** |  | 1^a^ | 0.21^b^ | 0.24^b^ |

1. χ^2^ – test, df=1 b. t-test ,df=143

**Table S2** Basic information on the BrainSpan Dataset

| **Regions** | **Tissues** | **Number of samples** | | |
| --- | --- | --- | --- | --- |
|  |  | 8pcw- 37pcw | 4mos- 8yrs | 13yrs- 40yrs |
| **FC** | Dorsolateral prefrontal cortex | 17 | 10 | 8 |
|  | Anterior (rostral) cingulate (medial prefrontal) cortex | 15 | 9 | 8 |
|  | Orbital frontal cortex | 14 | 8 | 9 |
|  | Ventrolateral prefrontal cortex | 16 | 10 | 9 |
| **SC** | Striatum | 14 | 6 | 8 |
|  | Mediodorsal nucleus of thalamus | 8 | 8 | 8 |
|  | Amygdaloid complex | 14 | 10 | 9 |
|  | Hippocampus | 15 | 8 | 9 |
| **SM** | Primary auditory cortex (core) | 14 | 8 | 9 |
|  | Primary motor cortex (area M1, area 4) | 10 | 7 | 9 |
|  | Primary somatosensory cortex (areas S1,3,1,2) | 10 | 7 | 9 |
|  | Primary visual cortex (striate cortex, area V1/17) | 15 | 10 | 8 |
| **TP** | Inferolateral temporal cortex (area TEv, area 20) | 13 | 11 | 10 |
|  | Posterior (caudal) superior temporal cortex (area TAc) | 14 | 12 | 10 |
|  | Posteroinferior (ventral) parietal cortex | 14 | 9 | 10 |

Four anatomic regions—frontal cortex (FC), subcortical regions (SC), sensory-motor regions (SM); and temporal and parietal regions (TP).

Three brain developmental stages— fetal (1: 8–37 post-conception weeks, pcw), early infancy to late childhood (2: 4 months to 8 years), and adolescence to adulthood (3: 13–40 years).

**Table S3** Top 20 significant SNPs from the voxel-wise association study (The full table of significant SNPs can be found in Table S7)

| **SNP ID** | **Gene** | ***p-*value** | **t-stat^a^** | **X^b^** | **Y^b^** | **Z^b^** | **AAL Region** | **MAF^c^** |
| --- | --- | --- | --- | --- | --- | --- | --- | --- |
| **rs10059334** | *PDE4D* | 2.56E-09 | 6.37 | -34 | 27 | -25 | OFClat_L | 0.062 |
| **rs2645810** | *INPP4B* | 3.32E-09 | -6.32 | -46 | 13 | 43 | Frontal_Mid_L | 0.079 |
| **rs3792299** | *EIF2B5* | 6.22E-09 | 6.19 | 10 | -4 | 55 | Supp_Motor_Area_R | 0.136 |
| **rs907094** | *PPP1R1B* | 8.25E-09 | 6.13 | -9 | -42 | 52 | Precuneus_L | 0.421 |
| **rs3764352** | *PPP1R1B* | 8.25E-09 | 6.13 | -9 | -42 | 52 | Precuneus_L | 0.421 |
| **rs2102996** | *ERBB4* | 1.25E-08 | 6.05 | 43 | -16 | 18 | Rolandic_Oper_R | 0.29 |
| **rs4926249** | *CACNA1A* | 1.42E-08 | -6.02 | 37 | -31 | 13 | Temporal_Sup_R | 0.41 |
| **rs7714765** | *PDE4D* | 1.54E-08 | 6.01 | -34 | 27 | -25 | OFClat_L | 0.062 |
| **rs6671271** | *NOS1AP* | 1.63E-08 | 6 | -24 | -60 | 69 | Parietal_Sup_L | 0.292 |
| **rs10994198** | *ANK3* | 1.65E-08 | 5.99 | -10 | -73 | 40 | Precuneus_L | 0.179 |
| **rs13008919** | *NRXN1* | 1.76E-08 | -5.98 | -33 | 14 | 40 | Frontal_Mid_L | 0.372 |
| **rs4682165** | *ZBTB20* | 1.92E-08 | -5.96 | 30 | -84 | -15 | Occipital_Inf_R | 0.205 |
| **rs2645813** | *INPP4B* | 2.33E-08 | -5.92 | -46 | 13 | 43 | Frontal_Mid_L | 0.076 |
| **rs4926248** | *CACNA1A* | 2.67E-08 | -5.9 | 45 | -25 | 22 | Rolandic_Oper_R | 0.41 |
| **rs336361** | *INPP4B* | 3.05E-08 | 5.87 | 40 | -51 | 60 | Parietal_Sup_R | 0.072 |
| **rs1918935** | *MAGI2* | 3.38E-08 | 5.85 | -36 | -61 | 33 | Angular_L | 0.135 |
| **rs9817665** | *ZBTB20* | 3.49E-08 | -5.84 | -54 | -37 | 46 | Parietal_Inf_L | 0.2 |
| **rs627428** | *GRIK2* | 3.58E-08 | -5.83 | 42 | 38 | 21 | Frontal_Mid_R | 0.073 |
| **rs7429036** | *EIF2B5* | 3.83E-08 | -5.82 | 21 | -1 | -43 | Fusiform_R | 0.387 |
| **rs7618415** | *ATP2B2* | 3.87E-08 | 5.82 | -51 | 15 | -10 | Temporal_Pole_Sup_L | 0.055 |

1. t-test ,df=140; b. Talairach coordinate; c. MAF: minor allele frequency.

**Table S4** The *p*-values of the interconnectedness of the 108 candidate genes in the spatial-temporal brain developmental gene networks under different construction strategies (“w” denotes the weighted networks)

| Region &Period | P-0.6w | P-0.6 | p-0.7w | p-0.7 | p-0.8w | p-0.8 | p-|PCC| |
| --- | --- | --- | --- | --- | --- | --- | --- |
| FC1 | 0.1896 | 0.2152 | 0.124 | 0.1305 | 0.0759 | 0.0792 | 0.1492 |
| FC2 | 4.00E-04 | 1.00E-04 | 5.00E-04 | 5.00E-04 | 4.00E-04 | 4.00E-04 | 3.00E-04 |
| FC3 | 0.001 | 0.0015 | 7.00E-04 | 8.00E-04 | 0.0011 | 7.00E-04 | 4.00E-04 |
| SC1 | 0.6186 | 0.5925 | 0.6832 | 0.6532 | 0.8931 | 0.8787 | 0.3575 |
| SC2 | 0.0125 | 0.0092 | 0.0191 | 0.0188 | 0.0599 | 0.0585 | 0.0032 |
| SC3 | 0.0271 | 0.0213 | 0.0828 | 0.0608 | 0.603 | 0.5737 | 0.0106 |
| SM1 | 0.3239 | 0.3324 | 0.2873 | 0.2872 | 0.2615 | 0.2692 | 0.4063 |
| SM2 | 1.00E-04 | 6.00E-04 | 5.00E-04 | 3.00E-04 | 5.00E-04 | 2.00E-04 | 5.00E-04 |
| SM3 | 0 | 0 | 0 | 1.00E-04 | 1.00E-04 | 0 | 1.00E-04 |
| TP1 | 0.4053 | 0.3645 | 0.6055 | 0.5795 | 0.8329 | 0.8221 | 0.426 |
| TP2 | 3.00E-04 | 2.00E-04 | 2.00E-04 | 6.00E-04 | 0.0037 | 0.0031 | 4.00E-04 |
| TP3 | 8.00E-04 | 8.00E-04 | 3.00E-04 | 6.00E-04 | 0.0014 | 8.00E-04 | 6.00E-04 |
| Merged | 0.4229 | 0.4244 | 0.3839 | 0.3753 | 0.4294 | 0.4179 | 0.2338 |

The 12 spatial-temporal networks were corresponding to 4 anatomic regions—frontal cortex (FC), subcortical regions (SC), sensory-motor regions (SM); and temporal and parietal regions (TP); and combined with 3 brain developmental stages— fetal (1: 8–37 post-conception weeks), early infancy to late childhood (2: 4 months to 8 years), and adolescence to adulthood (3: 13–40 years). |PCC|: absolute value of Pearson correlation coefficient.

**Table S5** The 16 hot-clusters identified (ranked according to the “Maximum Peak” in the last column)

| **Cluster ID** | **Associated Gene** | **AAL Region** | **Brain Region^a^** | | **Cluster Size** | | **Mean GMV** | **X^b^** | **Y^b^** | **Z^b^** | **Max**  **Weight** |
| --- | --- | --- | --- | --- | --- | --- | --- | --- | --- | --- | --- |
| **HC1** | CACNA1A  ERBB4 | Insula_R | | SM | 945 | 0.48 | | 40 | -28 | 16 | 0.69 |
| **HC2** | PPP1R1B | Precuneus_L | | TP | 438 | 0.54 | | -10 | -40 | 51 | 0.59 |
| **HC3** | ATP2B2 | Temporal_Pole_Sup_L | | TP | 118 | 0.26 | | -51 | 13 | -10 | 0.39 |
| **HC4** | PPP3CA  NRG3 | Temporal_Pole_Mid_R | | TP | 134 | 0.24 | | 36 | 15 | -40 | 0.38 |
| **HC5** | ZBTB20  RELN | Precentral_R | | SM | 157 | 0.4 | | 49 | -7 | 42 | 0.37 |
| **HC6** | OPCML | Supra Marginal_R | | TP | 74 | 0.28 | | 60 | -43 | 27 | 0.35 |
| **HC7** | INPP4B | Frontal_Mid_L | | FC | 63 | 0.99 | | -46 | 13 | 45 | 0.33 |
| **HC8** | ZNF365 | Temporal_Mid_R | | TP | 44 | 0.78 | | 52 | -72 | 15 | 0.31 |
| **HC9** | ANK3 | Precuneus_L | | TP | 47 | 0.26 | | -10 | -73 | 39 | 0.31 |
| **HC10** | ZBTB20 | Parietal_Inf_L | | TP | 31 | 0.51 | | -54 | -39 | 46 | 0.3 |
| **HC11** | PSAP | Lingual_L | | OC | 33 | 0.36 | | -10 | -67 | 1 | 0.29 |
| **HC12** | BMP6  EIF2B5 | Supp_Motor_Area_R | | SM | 20 | 0.47 | | 9 | -4 | 55 | 0.28 |
| **HC13** | NRG3  PRKCA | Precuneus_R | | TP | 16 | 0.5 | | 3 | -54 | 45 | 0.27 |
| **HC14** | FGF1 | Angular_L | | TP | 11 | 0.24 | | -43 | -52 | 24 | 0.26 |
| **HC15** | SLIT3 | Cingulate_Mid_R | | FC | 8 | 0.24 | | 12 | -18 | 45 | 0.26 |
| **HC16** | GFRA2 | Hippocampus_R | | SC | 11 | 0.24 | | 33 | -31 | -7 | 0.26 |

More detailed table on the hot clusters and HC genes. Column 6 shows the mean GMV of each HC. Columns 7-9 give the Talairach coordinates of the centroid of each HC. The last column gives the maximum weight of voxels within each HC.

1. frontal cortex (FC), temporal and parietal regions (TP), sensory-motor regions (SM), subcortical regions (SC) and Occipital regions (OC);
2. Talairach coordinate.

**Table S6** Grey matter volume difference of hot clusters

|  | Group1 | Group2 | Group3 |
| --- | --- | --- | --- |
| HC1 | 8.46E-01 | 4.37E-01 | 5.29E-01 |
| HC2 | 2.07E-02 | 9.55E-01 | 2.63E-02 |
| HC3 | 1.14E-02 | 7.61E-02 | 6.38E-01 |
| HC4 | 1.87E-01 | 8.84E-01 | 2.88E-01 |
| HC5 | 4.67E-01 | 8.85E-01 | 6.10E-01 |
| HC6 | 8.51E-04^*^ | 1.46E-01 | 1.20E-01 |
| HC7 | 7.79E-01 | 7.28E-01 | 5.29E-01 |
| HC8 | 2.51E-02 | 1.69E-01 | 5.38E-01 |
| HC9 | 1.22E-03^*^ | 1.00E+00 | 2.29E-03^*^ |
| HC10 | 3.60E-01 | 5.40E-01 | 8.48E-01 |
| HC11 | 2.83E-01 | 5.07E-01 | 8.88E-02 |
| HC12 | 7.35E-01 | 5.48E-01 | 7.56E-01 |
| HC13 | 6.71E-01 | 4.91E-01 | 2.61E-01 |
| HC14 | 7.20E-03 | 1.49E-14^*^ | 4.40E-06^*^ |
| HC15 | 9.93E-04^*^ | 2.68E-01 | 6.03E-02 |
| HC16 | 3.76E-01 | 6.82E-01 | 7.06E-01 |

The *p-*values of a two sided Wilcoxon rank sum test of GMV with one group and the remaining patients. The threshold was selected as *p*-values < 0.05/16. The cells passing the threshold are shown in red.

**Figure S1** Slice views of the top 4 significant SNPs and genes with their associated location in brain.

1. *EIF2B5*: rs3792299
2. *INPP4B*: rs2645810


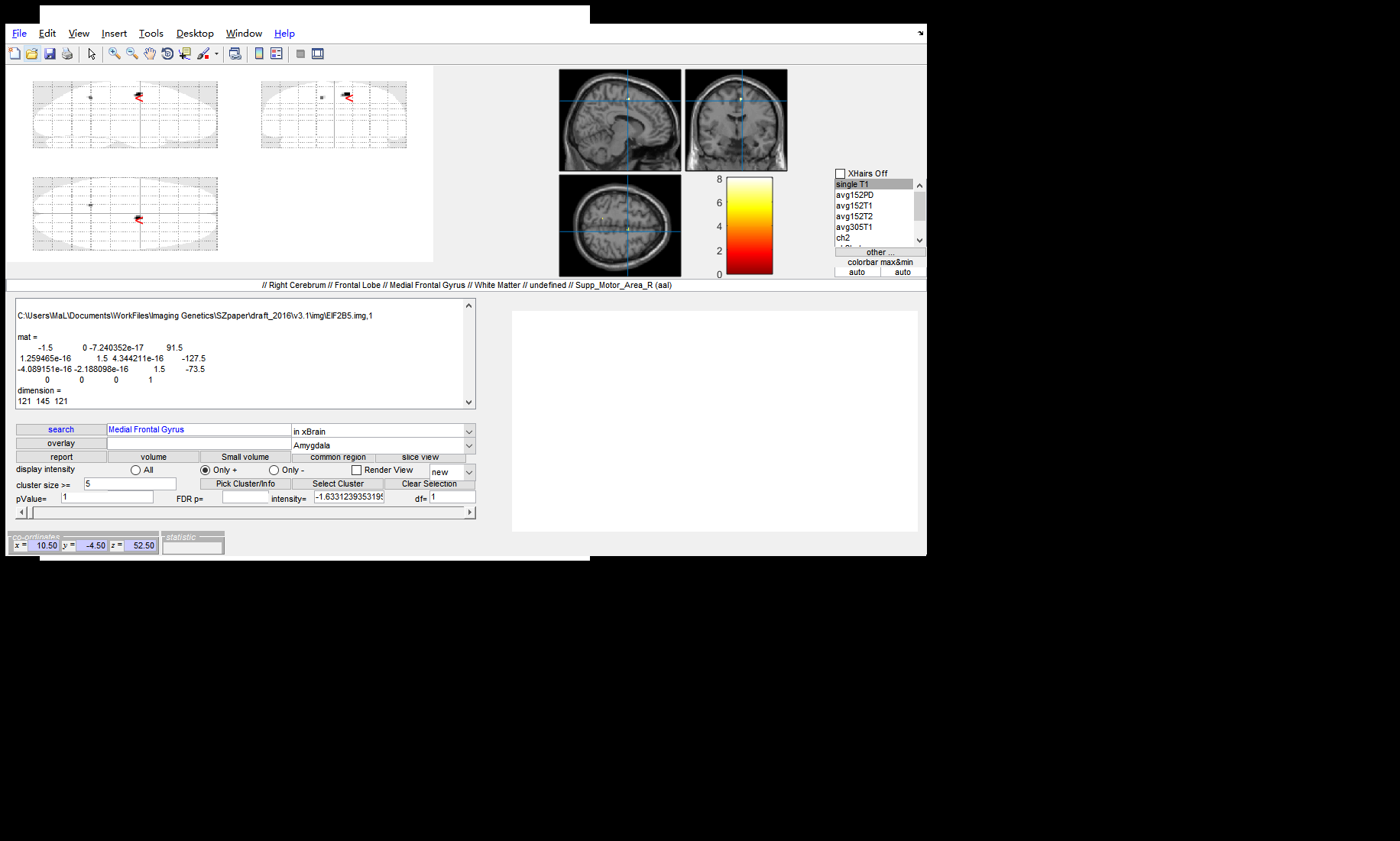

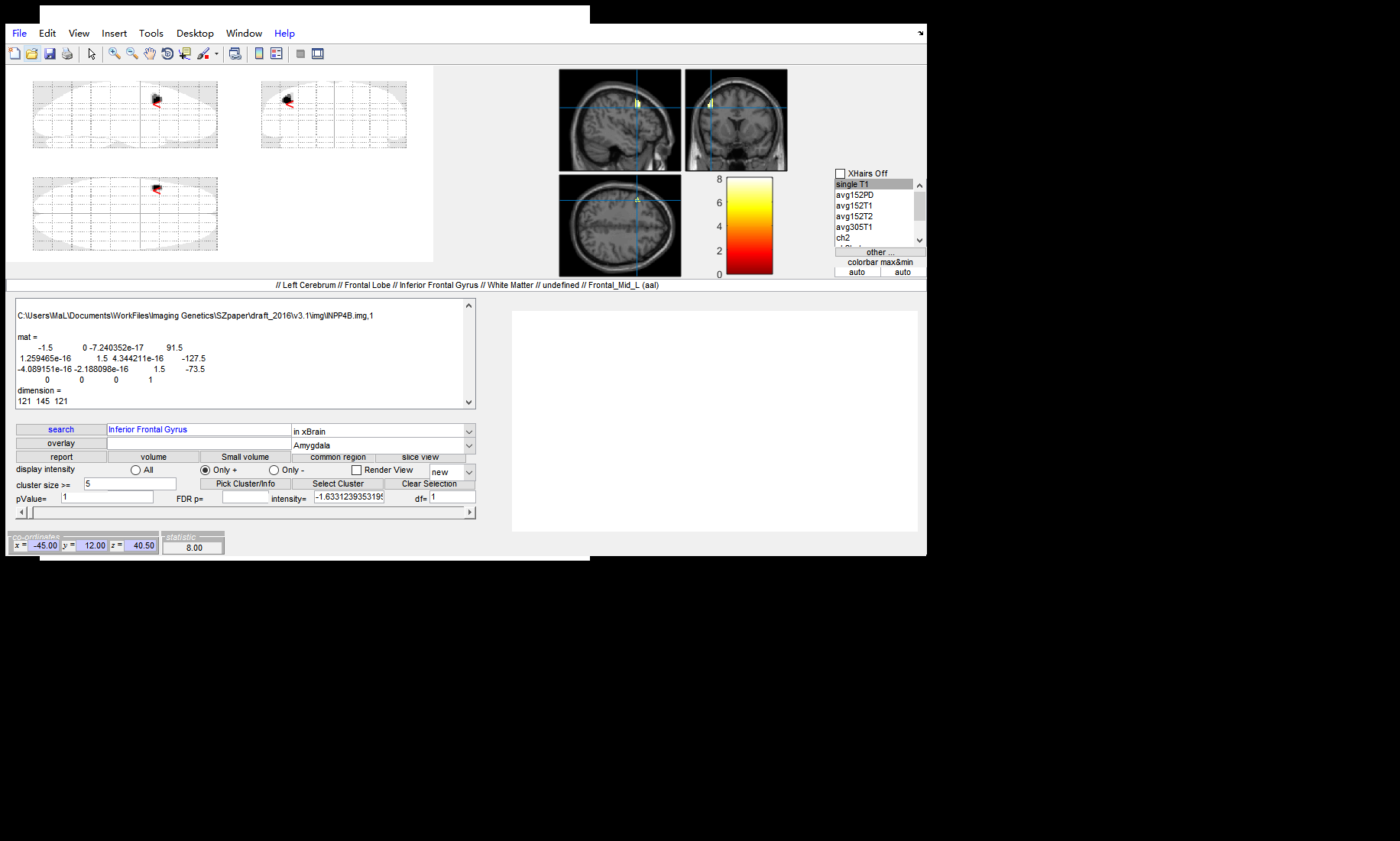

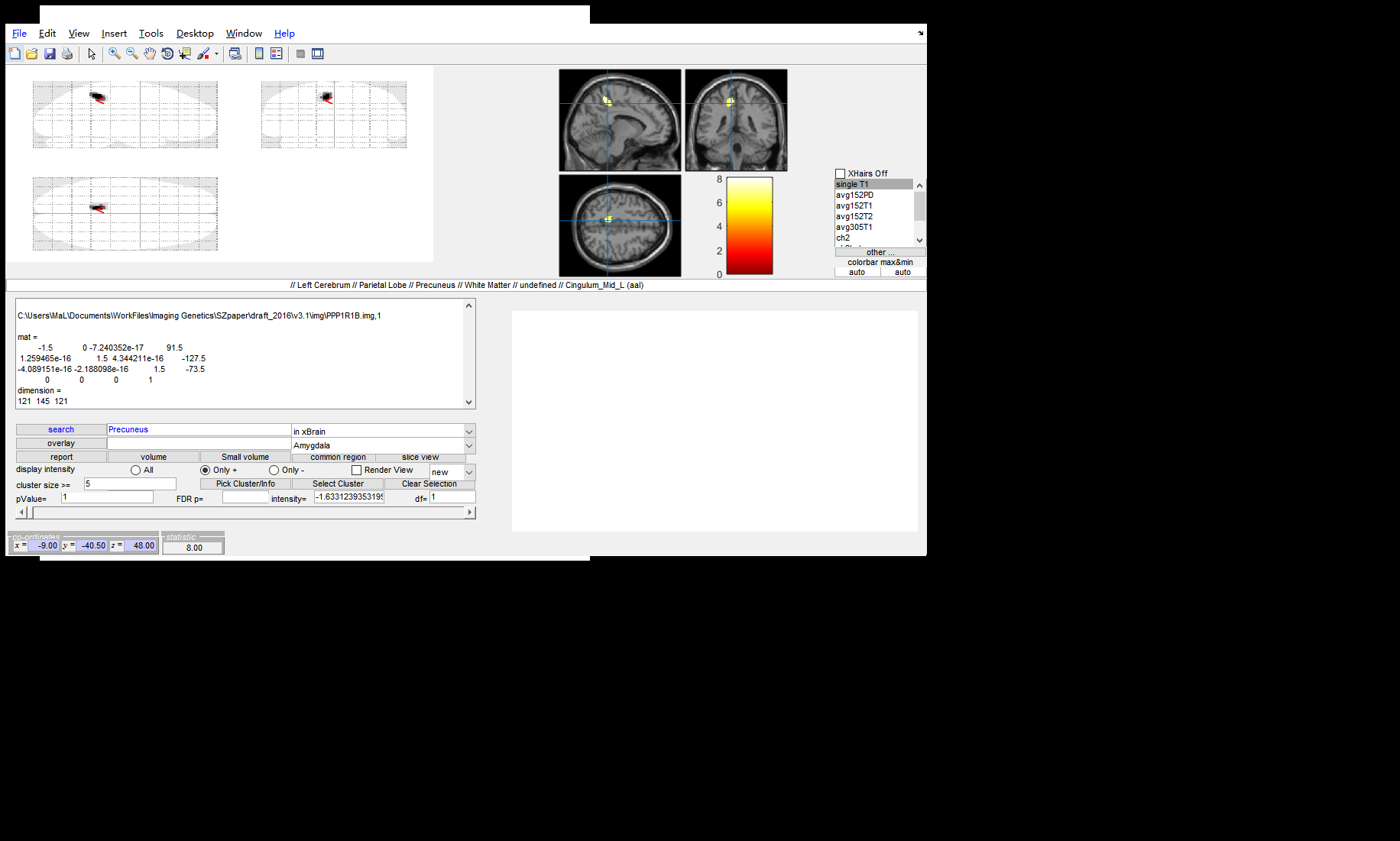

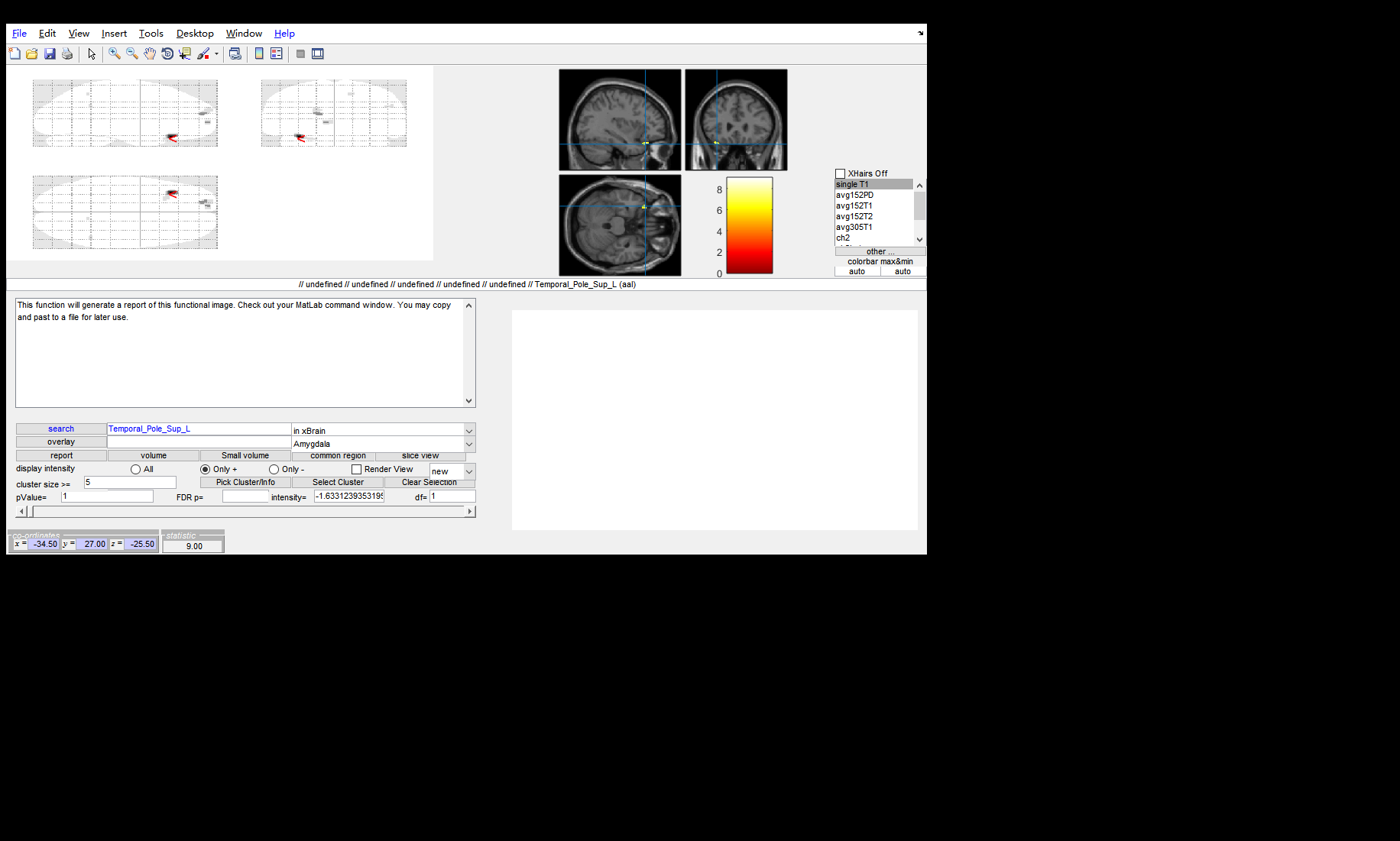


1. *PDE4D*: rs10059334
2. *PPP1R1B*: rs907094,

rs3764352

Slice views of the top 4 significant SNPs and genes with their associated location in brain. The intensity represents the logarithm transformation of the *p-*value of the corresponding SNPs in the linear regression model.

A. rs10059334 of gene *PDE4D* (minmum *p-*value:2.56E-09);

B. rs2645810 of gene *INPP4B* (minmum *p-*value: 3.32E-09);

C. rs3792299 of gene *EIF2B5* (minmum *p-*value: 6.22E-09);

D. rs3764352 and rs907094 of gene *PPP1R1B* (minmum *p-*value: 8.25E-09).

**Figure S2** Coronal views of the 16 hot clusters (HCs)


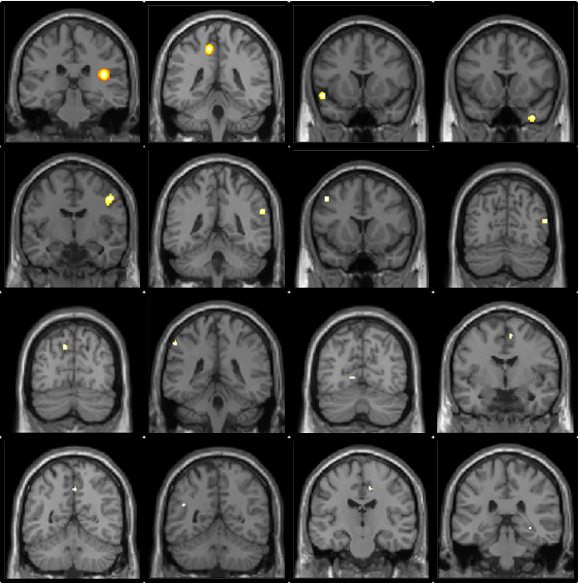

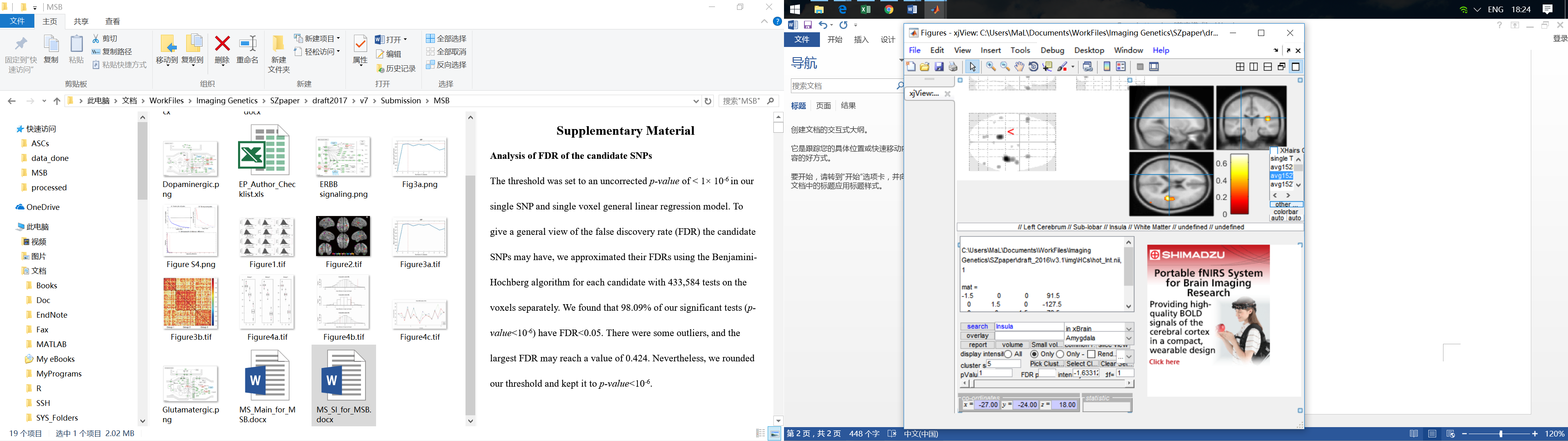


The intensity represents the corrected weight of each voxel. The HCs in the table are:

| HC1 | HC2 | HC3 | HC4 |
| --- | --- | --- | --- |
| HC5 | HC6 | HC7 | HC8 |
| HC9 | HC10 | HC11 | HC12 |
| HC13 | HC14 | HC15 | HC16 |

**Figure S3** The hot cluster genes in within pathways

**A**

**
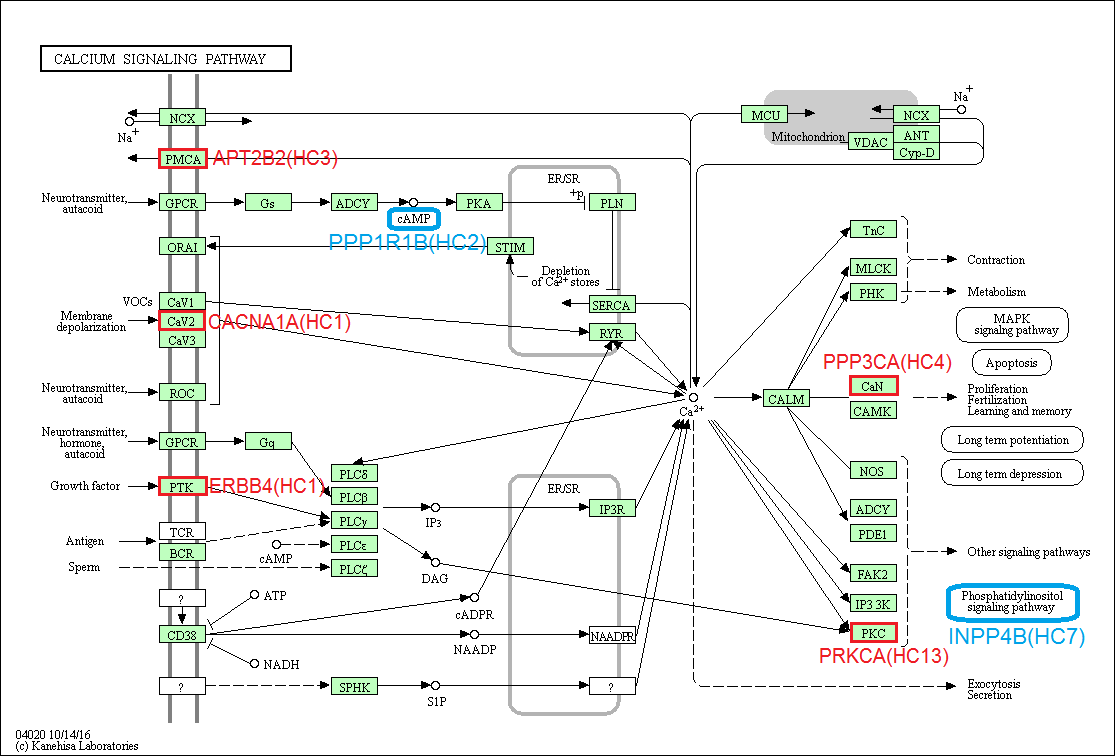
**

**B**


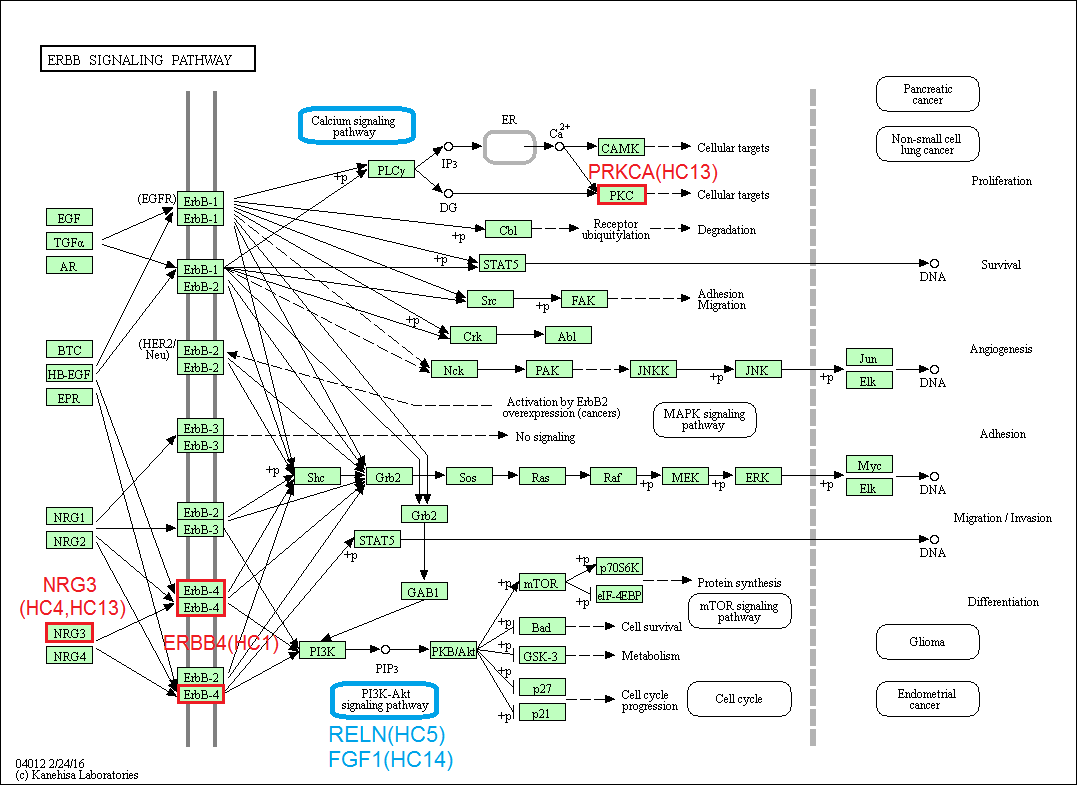


**C**

**
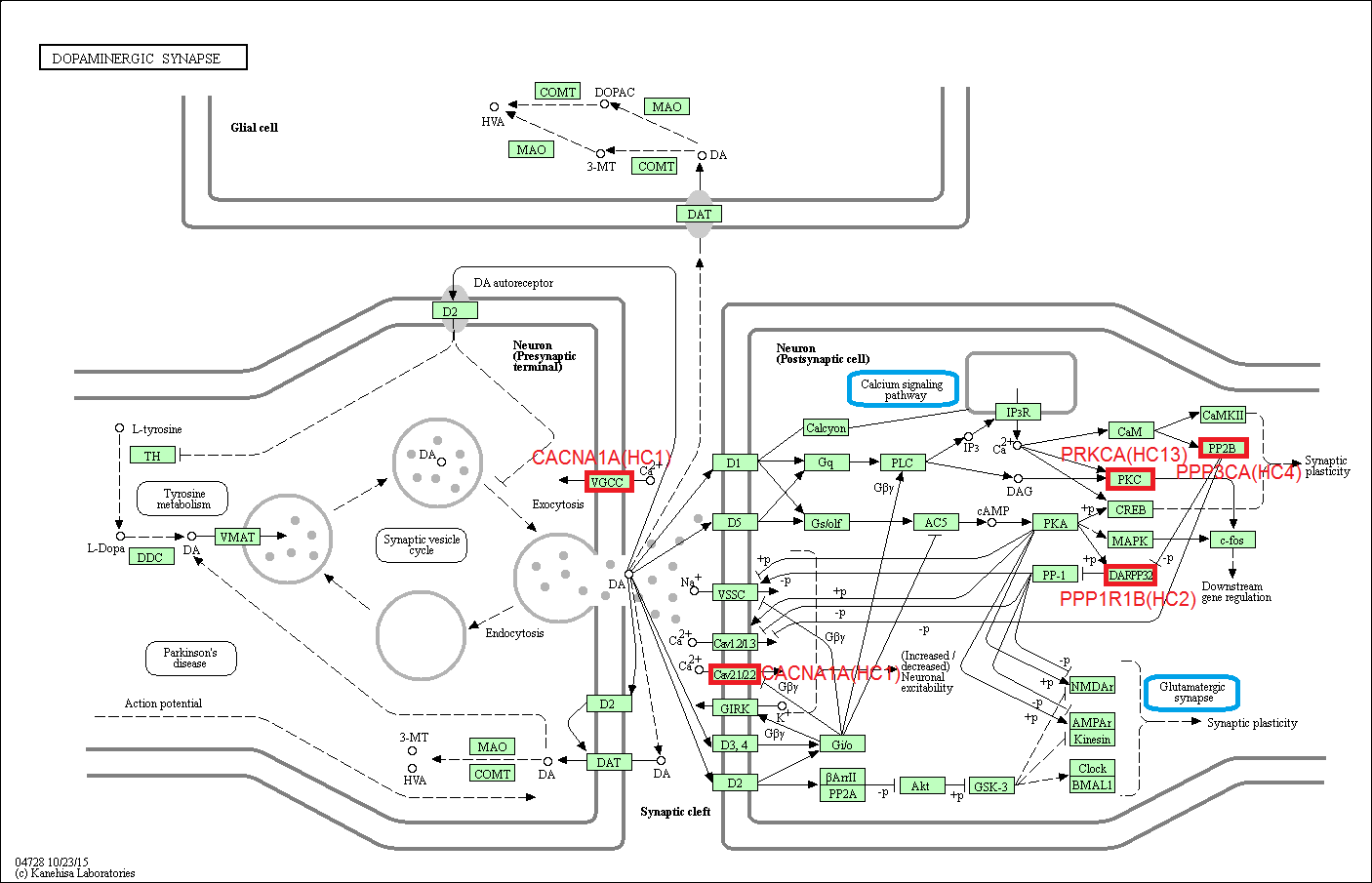
**

**D**


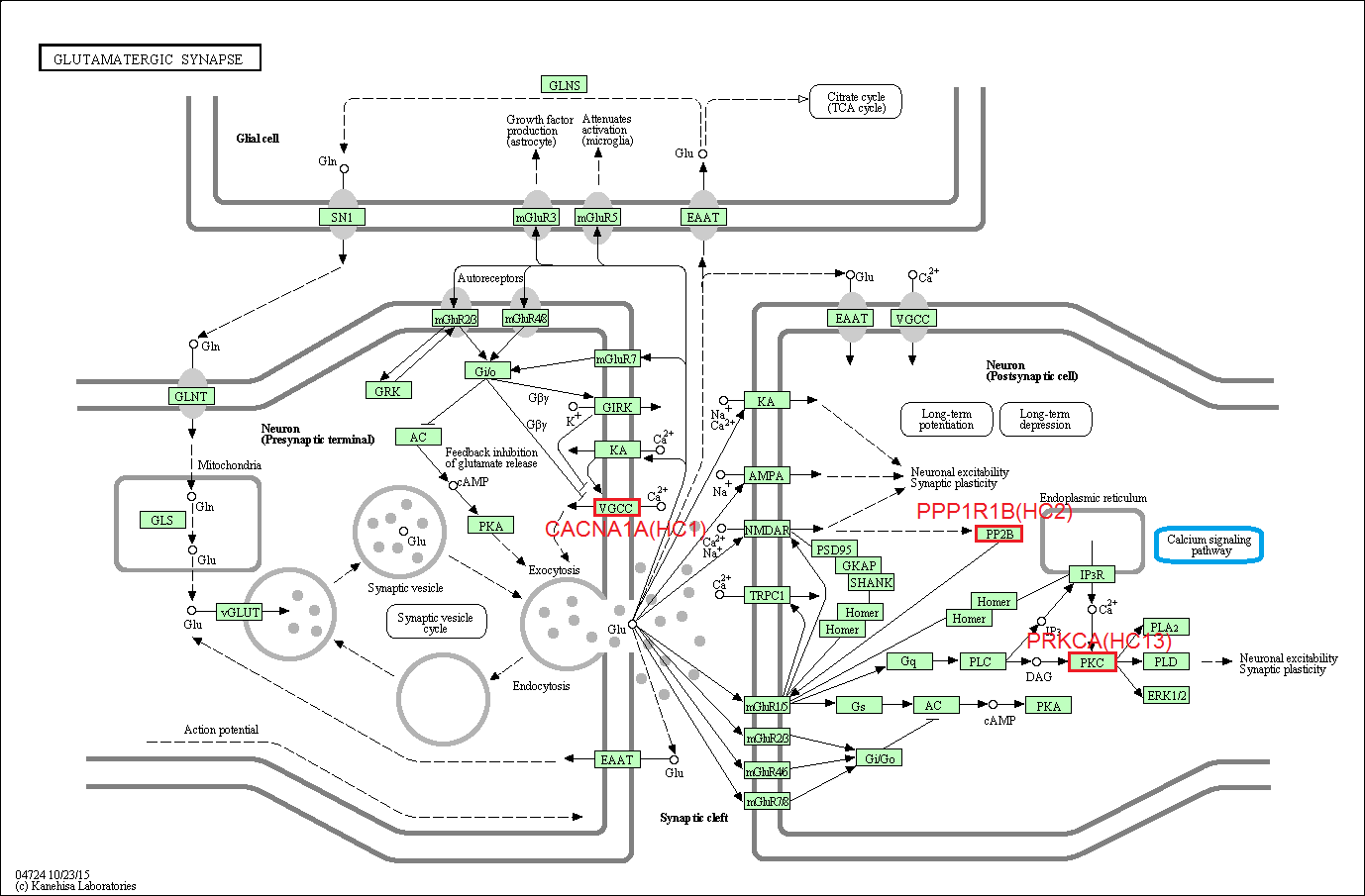


The pathway maps are adapted from KEGG (http://www.genome.jp/ kegg/). The HC genes were annotated in red. Pathways upstream or downstream that include HC genes were annotated in blue.

A. Calcium signal pathway.

B. ERBB signal pathway.

C. Dopaminergic synapse pathway.

D. Glutamatergic synapse pathway.

**Figure S4** PANSS of different subtyped patient groups

A


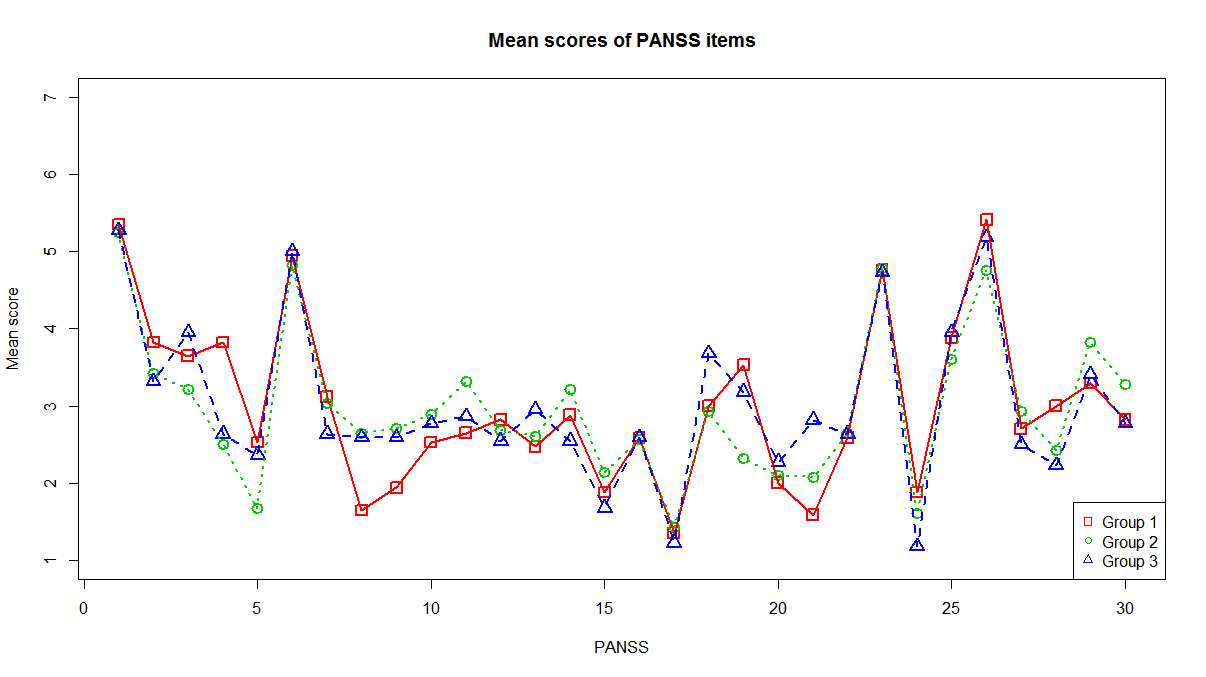


B


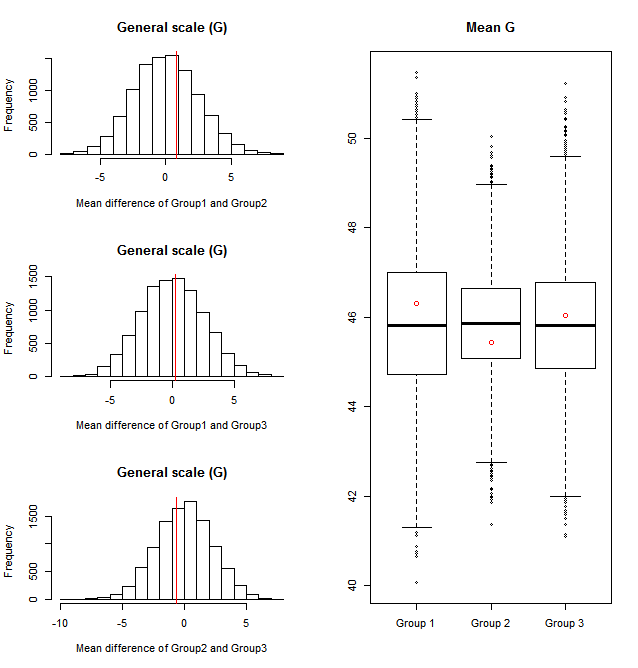


C


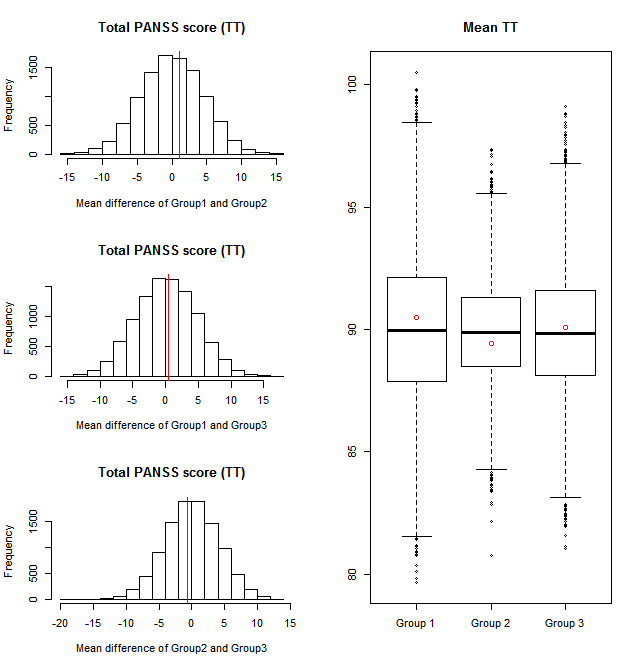


The histogram shows the pairwise difference of mean scores between two groups, with the red line representing the true difference. The actual means of each group are shown in red. Permutation tests of means of general PANSS score and total PANSS score. The box plots show the distribution of mean scores of each group over the 10000 permutation runs, with red circle indicating the actual mean.

1. The mean group scores of each PANSS item, with Group 1 shown in red, Group 2 in green and Group 3 in blue.
2. The mean difference of the general PANSS scale scores between groups 1 and 2 (MeanG12), groups 1 and 3 (MeanG13), and groups 2 and 3 (MeanG23) over 10000 permutations are shown in histograms. The x-axis represent the difference of mean scores of a pair of permutated groups. The quantile of the *p-value* is represented by red lines. The distributions based on permutation of the group mean scores of the general PANSS scores are shown in the boxplot.
3. The mean difference of the total PANSS score between groups 1 and 2 (MeanG12), groups 1 and 3 (MeanG13), groups 2 and 3 (MeanG23) over 10000 permutations are shown in histograms. The x-axis represent the difference of mean scores of a pair of permuted groups. The quantile of *p-value* is represented by red lines. The distributions based on permutation of the group mean scores of the general PANSS core are shown in the boxplot.

**Figure S5** FDR distribution of candidate SNPs


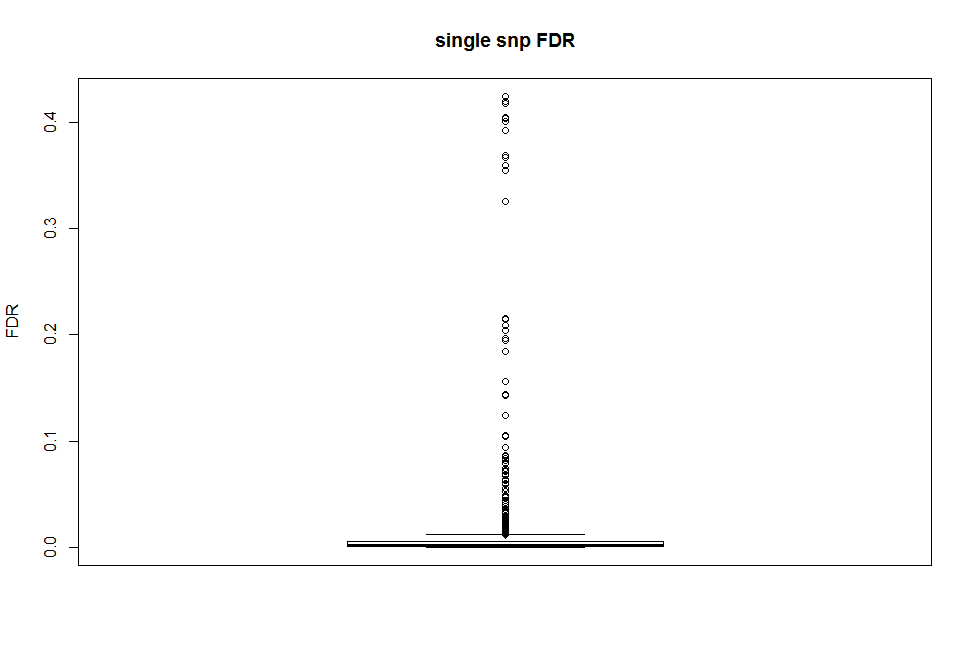


Boxplot of the FDRs for test(s) with *p-value*<10^-6^ calculated for each candidate SNP separately.

**Figure S6** Thresholding weights of voxels


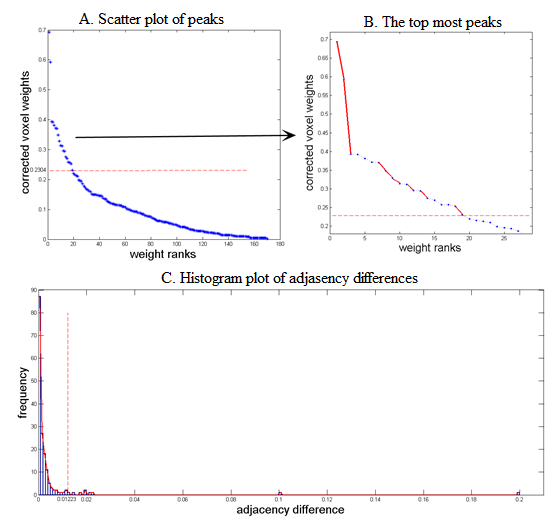


A. The corrected voxel weights VS weight ranks. The red dashed line shows the cluster forming threshold.

B. An enlarged version of plot (a). The red lines between points indicate the top 5% adjacency differences.

C. Histogram plots of adjacency difference between the ranked weights of the peak voxels. The vertical red dashed line shows the 95% quantile of the differences.
